# Current intensity‐ and polarity‐specific online and aftereffects of transcranial direct current stimulation: An fMRI study

**DOI:** 10.1002/hbm.24901

**Published:** 2019-12-20

**Authors:** Asif Jamil, Giorgi Batsikadze, Hsiao‐I. Kuo, Raf L. J. Meesen, Peter Dechent, Walter Paulus, Michael A. Nitsche

**Affiliations:** ^1^ Department Psychology and Neurosciences Leibniz Research Centre for Working Environment and Human Factors Dortmund Germany; ^2^ REVAL Research Institute, University of Hasselt Hasselt Belgium; ^3^ Department of Neurology, Essen University Hospital University of Duisburg‐Essen Essen Germany; ^4^ Department of Cognitive Neurology University Medical Center, University of Göttingen Göttingen Germany; ^5^ Department of Clinical Neurophysiology University Medical Center, University of Göttingen Göttingen Germany; ^6^ Department of Neurology University Medical Hospital Bergmannsheil Bochum Germany

**Keywords:** arterial spin labeling, cerebral blood flow, current intensity, inter‐individual variability, motor cortex, transcranial direct current stimulation

## Abstract

Transcranial direct current stimulation (tDCS) induces polarity‐ and dose‐dependent neuroplastic aftereffects on cortical excitability and cortical activity, as demonstrated by transcranial magnetic stimulation (TMS) and functional imaging (fMRI) studies. However, lacking systematic comparative studies between stimulation‐induced changes in cortical excitability obtained from TMS, and cortical neurovascular activity obtained from fMRI, prevent the extrapolation of respective physiological and mechanistic bases. We investigated polarity‐ and intensity‐dependent effects of tDCS on cerebral blood flow (CBF) using resting‐state arterial spin labeling (ASL‐MRI), and compared the respective changes to TMS‐induced cortical excitability (amplitudes of motor evoked potentials, MEP) in separate sessions within the same subjects (*n* = 29). Fifteen minutes of sham, 0.5, 1.0, 1.5, and 2.0‐mA anodal or cathodal tDCS was applied over the left primary motor cortex (M1) in a randomized repeated‐measure design. Time‐course changes were measured before, during and intermittently up to 120‐min after stimulation. ROI analyses indicated linear intensity‐ and polarity‐dependent tDCS after‐effects: all anodal‐M1 intensities increased CBF under the M1 electrode, with 2.0‐mA increasing CBF the greatest (15.3%) compared to sham, while all cathodal‐M1 intensities decreased left M1 CBF from baseline, with 2.0‐mA decreasing the greatest (−9.3%) from sham after 120‐min. The spatial distribution of perfusion changes correlated with the predicted electric field, as simulated with finite element modeling. Moreover, tDCS‐induced excitability changes correlated more strongly with perfusion changes in the left sensorimotor region compared to the targeted hand‐knob region. Our findings reveal lasting tDCS‐induced alterations in cerebral perfusion, which are dose‐dependent with tDCS parameters, but only partially account for excitability changes.

## 1 INTRODUCTION

Modulation of cortical neuroplasticity in humans—the process responsible for learning, memory and repair—stands as a critical learning objective in the fields of clinical neurology and cognitive neuroscience. Classic techniques, such as the use of extracellular stimulation and recording electrodes in animal models and pharmacological modulation of central neurotransmitters in human models, have revealed substantial insights into mechanisms of long‐term plasticity, such as the fundamental role of the synaptic glutamatergic system in inducing long‐term potentiation (LTP) or long‐term depression (LTD) (Bliss, Cooke, Ii, & Cooke, 2011; Cooke & Bliss, 2006; Lüscher & Malenka, 2012; Rowland et al., 2005; Tahar, Blanchet, & Doyon, 2004). Moreover, the recent development of noninvasive brain stimulation methods has provided the capability to bi‐directionally modulate and probe these alterations at a system level in a safe and controlled manner (Bikson et al., 2016; Huang, Lu, et al., 2017; Stefan, Kunesch, Cohen, Benecke, & Classen, 2000; Ziemann et al., 2008). One of the foremost techniques is transcranial direct current stimulation (tDCS), which has shown potential as it is inexpensive, well‐tolerated, and suitable for a wide range of applications, such as in modulation of cognitive processes in healthy humans, but also across clinical scenarios, such as stroke rehabilitation or alleviation of depression (Fresnoza et al., 2014; Gandiga, Hummel, & Cohen, 2006; Nitsche, Boggio, Fregni, & Pascual‐Leone, 2009; Polanía, Nitsche, & Ruff, 2018).

tDCS is based on the DC application of a subthreshold neuronal electric field lasting for several minutes, and is usually delivered through two or more conductive electrodes placed on the scalp, with current intensities ranging between 1–2 mA (Woods et al., 2016). Seminal studies in animal models demonstrated that very weak electric fields are sufficient to modulate the spontaneous firing rates of neurons for up to several hours, which are stimulation polarity dependent (Bindman, Lippold, & Redfearn, 1962; Creutzfeldt, Fromm, & Kapp, 1962; Terzuolo & Bullock, 1956). More recent human pharmacology studies combined with transcranial magnetic stimulation (TMS) over the primary motor cortex (M1) have elaborated on the physiological determinants of stimulation effects. Primary or acute effects depend on activity of voltage‐gated sodium and calcium channels, which induce de‐ or hyper‐polarization of the resting membrane potential, thereby regulating the spontaneous neuronal firing rate, and cortical excitability measured using the TMS motor evoked potential (MEP) (Liebetanz, Nitsche, Tergau, & Paulus, 2002; Nitsche, Fricke, et al., 2003; Nitsche et al., 2004). When stimulation duration is extended from seconds to multi‐minutes, longer lasting after‐effects in excitability are observed, lending to a notion that dependencies of stimulation intensity, polarity, and duration share mechanistic properties of long‐term potentiation (LTP) and long‐term depression (LTD) models of synaptic plasticity (Hattori, Moriwaki, & Hori, 1990; Islam, Aftabuddin, Moriwaki, Hattori, & Hori, 1995; Islam, Moriwaki, et al., 1995; Nitsche, Nitsche, et al., 2003; Nitsche & Paulus, 2001). Furthermore, pharmaco‐TMS, and magnetic resonance spectroscopy studies demonstrated the dependence of both anodal and cathodal tDCS‐induced LTP‐ and LTD‐like plasticity from NMDA‐, as well as GABA‐receptor activity (Liebetanz et al., 2002; Nitsche, Fricke, et al., 2003; Stagg, Best, et al., 2009).

Although these multi‐minute tDCS protocols have been widely adopted for research and clinical applications, the extent of the interaction between tDCS parameters and the physiological activity and mechanisms underlying prolonged effects has not yet been fully clarified. For example, duration of tDCS after‐effects are generally influenced by the stimulation duration, current intensity, and the number of treatment sessions; however, the dose–response to manipulating these parameters (ex: by prolonging the stimulation duration (Monte‐Silva et al., 2013; Monte‐Silva, Kuo, Liebetanz, Paulus, & Nitsche, 2010), or increasing the current intensity (Batsikadze, Moliadze, Paulus, Kuo, & Nitsche, 2013; Jamil et al., 2017)) do not necessarily lead to greater effects. This nonlinearity is an important concern, as it indicates a need for more nuanced experimental approaches where stimulation parameters are investigated through systematic titrations (Esmaeilpour et al., 2018). Moreover, given the extant nature of physiological findings on areas other than the M1, an open question for development of clinical protocols is whether the physiological findings of tDCS‐MEP studies conducted in the M1 can be extrapolated in order to understand effects of tDCS in other cortical areas. This is not self‐evident considering that the mean MEP amplitude represents a complex measure of neuronal excitability not only local to the motor area, but also the premotor area, and also across several subpopulations of neurons in the cortical, subcortical, and spinal layers (Groppa et al., 2012). Thus, the effects of M1 stimulation as recorded using TMS‐MEP not only may not translate one‐to‐one to other cortical areas, but also may not be completely comparable with other modalities that record neuronal activity in areas other than the M1, such as neuroimaging methods. In this view, evidence for dose‐dependent physiological effects measured using imaging remains relatively scant. In an animal study using laser Doppler flowmetry (LDF) to address whether tDCS directly influences cerebral blood flow (CBF), Wachter et al. (2011) reported polarity‐dependent modulation, such that CBF increased following anodal tDCS lasting 30 min, while CBF decreased following cathodal tDCS. Using arterial spin labeling (ASL) to directly measure CBF in humans, Zheng, Alsop, and Schlaug (2011) reported a monotonic intensity‐dependent correlation between current intensity (0.8–2 mA) and CBF underneath the anode, although their monitoring was limited to include only online and short‐lasting after‐effects. Thus, it remains unknown to what extent after‐effects in CBF are intensity‐ and polarity‐dependent over a more prolonged period of time, which would reflect more accurately the dynamics of tDCS‐induced neuroplasticity, and not merely direct effects of DC current on vessel dilation (Berliner, 1997; Durand, Fromy, Bouye, Saumet, & Abraham, 2002), and whether after‐effects on CBF mirror those observed in cortical excitability.

The present study was designed to address the question of the dose–response relationship of tDCS on cortical physiology, specifically asking whether increasing the current‐intensity parameter between 0.5–2.0 mA of anodal and cathodal tDCS would result in physiologically linear effects, as might be predicted from previous studies with short‐duration protocols (Nitsche & Paulus, 2000) and computational models of the induced electric field (Datta et al., 2011). ASL‐fMRI was used to monitor changes in local and global resting state CBF during and up to 2 hr after the end of M1 stimulation. Importantly, in order to compare whether after‐effects in CBF are correlated with changes in TMS‐MEP, we re‐enrolled participants who took part in our recent TMS‐MEP study which investigated intensity and polarity‐dependent changes in cortical excitability using the same parameters (Jamil et al., 2017), thereby providing ourselves with a larger dataset which allowed us to assess subject‐level correlations between TMS‐MEP and fMRI‐CBF. We hypothesized that as tDCS induces polarity‐dependent after‐effects in cortical excitability, CBF would be modulated in a polarity‐dependent manner, considering the relationship between neurovascular coupling, synaptic plasticity, and cerebral blood flow (Attwell et al., 2010). As additional exploratory analyses, we investigated whether realistic modeling of the electric field could accurately predict the extent of the physiological effects (Opitz et al., 2015), and we also assessed whether inter‐individual differences in the structural brain anatomies of our subjects were significant covariates interacting with neuroplastic after‐effects, which could offer insight into understanding the known inter‐individual variability in the response to tDCS (Chew, Ho, & Loo, 2015; López‐Alonso, Cheeran, Río‐Rodríguez, & Fernández‐Del‐Olmo, 2014; Wiethoff, Hamada, & Rothwell, 2014).

## 2 MATERIALS AND METHODS

### 2.1 Subjects

Initially, 32 healthy and nonsmoking participants were recruited for the study. Two subjects dropped out during the course of the 10 sessions, and one subject's data set was excluded due to a failure to remain completely relaxed in the scanner, resulting in excessive head motion and leading to mislabeling of perfusion and physiologically misleading results. The final data were analyzed from a sample size of 29 participants (16 males, mean age 25.0 ± 4.4 years). Subjects were randomly divided into two experimental groups of stimulation polarity (anodal and cathodal) and were blinded to both polarity and intensity of the stimulation throughout the 10 sessions of the study. All subjects were right‐handed as assessed by the Edinburgh Handedness Inventory (Oldfield, 1971). Prior to taking part, each participant provided written informed consent and was screened by a medical professional to verify no history of neurological disease, not on active medication, not wearing metal implants and not pregnant. Twenty‐eight of the participants were naïve to tDCS. The study was approved by the Medical Ethics Committee of the University of Göttingen, and all subjects were compensated for their participation. The data that support the findings of this study are available on request from the corresponding author. The data are not publicly available due to privacy or ethical restrictions.

### 2.2 TMS measures

In order to associate changes in cerebral blood flow with changes in cortical excitability, we re‐enrolled participants who had taken part in a larger TMS study with the same tDCS parameters as here (for extended details, we invite readers to refer to (Jamil et al., 2017)).

### 2.3 MRI measures

MRI was conducted in a 3 Tesla Magnetom TrioTim (Siemens Healthcare, Erlangen, Germany) using a 32‐channel head coil. Stimulation electrodes were fitted before subjects were placed inside the magnet bore. Initially, anatomical images based on a T1‐weighted 3D turbo fast low angle shot (FLASH) MRI sequence at 1 mm^3^ isotropic resolution were recorded (repetition time (TR) 2,250 ms echo time (TE) 3.32 ms, inversion time 900 ms, flip angle 9°). Subsequent scans were divided in 10 blocks: prestimulation/baseline, stimulation, and then after‐effects measurements immediately as well as 15, 30, 45, 60, 75, 90, 105, and 120 min after stimulation. For each of the 10 blocks, three measurements were obtained: a resting‐state blood‐oxygen‐level‐dependent (BOLD) measurement (5 min 51 s), a resting‐state ASL measurement (5 min 8 s), and a gradient echo field mapping scan (1 min). The ordering of the ASL and BOLD scans was counter‐balanced evenly between subjects to mitigate any ordering effects. The analysis of the BOLD dataset was not considered within the scope of the current study.

ASL images were acquired using a pseudo‐continuous ASL (pcASL) sequence with the following parameters: TE 12 ms, TR 3750 ms, 24 slices, in‐plane resolution 3 × 3 mm, slice thickness 4 mm, 20% gap, flip angle 90, FOV 192 mm, labeling time 1,484 ms, postlabel delay 1 s, RF gap 360 us, RF blocks 80. Each ASL sequence was accompanied by a background‐suppressed proton density (PD) reference image using the same parameters, but without ASL labeling, which was used for functional registration and CBF calibration (see preprocessing, Section 2.5).

### 2.4 DC stimulation of the motor cortex

For both experiments, anodal and cathodal DC‐stimulation of the left motor cortex was performed using the same MR‐compatible constant‐current battery powered stimulator (neuroCare, Germany). The location of the target electrode on the scalp was determined individually for each subject by using the “motor hotspot” corresponding to the maximum TMS‐induced motor evoked potentials (MEPs) of the right‐hand ADM. The target electrode (35 cm^2^) was placed over the hotspot region, with a 45° rotation toward the midline (Foerster et al., 2018), and with the connector position oriented on the midline edge ((Saturnino, Antunes, & Thielscher, 2015) Figure 1b). The second electrode was made 10x10 cm^2^ in order to reduce the current density in the nontargeted region (Nitsche et al., 2007), and placed over the participant's right frontal orbit and with the connector position oriented toward the participant's right side. To reduce discomfort of the stimulation and to also ensure adequate blinding, a topical anesthetic cream consisting of 10% lidocaine (EMLA®, AstraZeneca, UK) was pre‐applied on the scalp electrode regions approximately 20 min prior to stimulation (Guleyupoglu, Febles, Minhas, Hahn, & Bikson, 2014; McFadden, Borckardt, George, & Beam, 2011). A layer of conductive paste (Ten20®, Weaver) was applied to each rubber electrode which provided the conductive medium. Based on the uniformly randomized ordering, anodal or cathodal tDCS at an intensity of 0.5, 1.0, 1.5, 2.0 mA or sham was delivered for 15 min, with a 10 s fade‐in and fade‐out at the beginning and end of stimulation. Fifteen minutes of stimulation is within the range of stimulation protocols producing polarity‐specific long‐term effects with 1 mA stimulation, with anodal tDCS enhancing, and cathodal tDCS reducing cortical excitability, without inducing late phase or converted effects (Batsikadze et al., 2013; Monte‐Silva et al., 2013), which would make interpretation of the results more complex. Changes in skin impedance during the stimulation was monitored and did not exceed 20 kΩ. For the sham condition, a DC intensity of 1.0 mA was delivered for 30 s, with a 20 s ramp, which has been shown to achieve effective stimulation blinding (Ambrus et al., 2012; Gandiga et al., 2006). In order to reduce electrode artifacts or eddy‐currents, care was taken to make sure electrodes exited across the right side of the participant, and through the back of the magnet bore without any twisting or loops. During fMRI blocks for which no DC stimulation was delivered, electrodes were kept disconnected from the stimulator (Antal et al., 2014).

**Figure 1:**
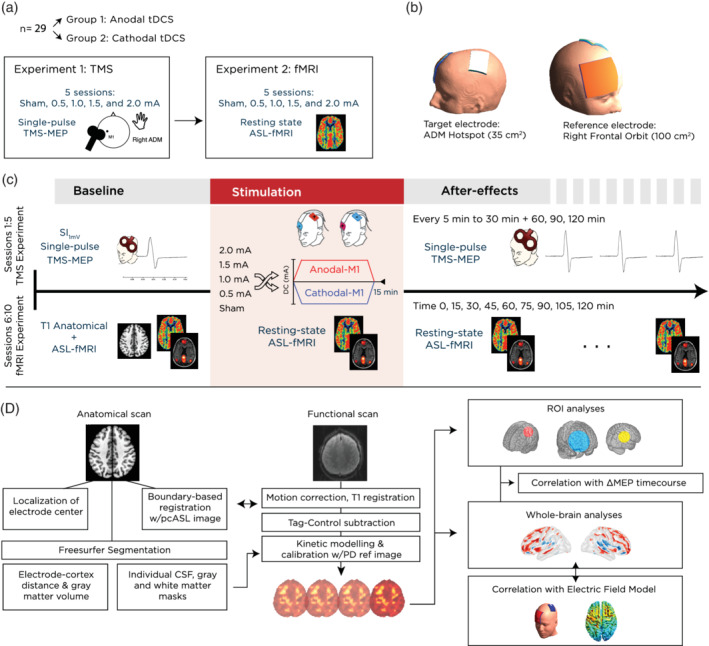
Experimental design and methods. (a) The study involved 29 participants divided into two groups, who took part in two consecutive experiments: a TMS‐based cortical excitability study to investigate the effect of current intensity on cortical excitability, and an fMRI study to investigate identical stimulation parameters on cerebral blood flow and functional connectivity. (b) Prior to the scanning session, the motor‐cortical representation of the right abductor digiti minimi muscle (ADM) was located using single‐pulse TMS. The respective position on the scalp was used to place a 35 cm^2^ target electrode, rotated 45° to the midline, and with the cable exiting from the right posterior edge. A larger 100 cm^2^ return electrode was positioned over the contralateral right orbit, with the cable exiting from the participant's right hand side. (c) Scanning sessions started with acquisition of a high resolution, T1‐weighted FLASH anatomical scan, followed by the first block of two resting state scans, consisting of either BOLD (6 min) or ASL (5 min) acquisitions (note that the ordering was counter‐balanced across subjects). This block was repeated an additional nine times, beginning with the stimulation block, where tDCS was delivered for 15 min using either sham, 0.5, 1.0, 1.5, or 2.0 mA anodal or cathodal stimulation. Subsequent measurements took place every 15 min following the stimulation, for up to 120 min. (d) The analysis pipeline included a separate preparation of anatomical images for extraction of indivdual antomical parameters, such as gray matter volume and electrode cortex distance. Functional images were preprocessed and registered to the subject's high resolution anatomical image, and then to the MNI template before proceeding with statistical analysis. ASL, arterial spin labeling; blood‐oxygen‐level‐dependent; tDCS, transcranial direct current stimulation; TMS, transcranial magnetic stimulation

### 2.5 Experimental procedures

#### 2.5.1 TMS experiment

We invite readers to refer to the accompanying study for comprehensive details of the TMS experiment (Jamil et al., 2017). Figure 1a displays an overview of the experimental procedure. Briefly, each subject underwent five experimental sessions (sham, 0.5, 1.0, 1.5, and 2.0 mA stimulation) in a pseudo‐randomized order (uniformly distributed, with a matrix generated using MATLAB), and where each session was separated by at least 7 days. Following a baseline measurement of 25 MEPs, 15 min of anodal or cathodal stimulation was delivered as previously described. After removal of tDCS electrodes, MEP measurements were taken immediately again in epochs of every 5 min up to 30 min after the stimulation, and then every 30 min up to 2 hr after stimulation (11 total epochs).

#### 2.5.2 fMRI experiment

Prior to beginning the scanning, the exact location of the ADM motor hotspot was recorded and marked using TMS. Subjects were then prepared with stimulation electrodes with the target electrode positioned over the hotspot area, and then situated comfortably inside the scanner. As a prospective effort to reduce head motion, a custom memory‐foam pillow was used in place of pads to secure the subjects head inside the MR receiving coil. An initial T1 anatomical scan was acquired, followed by the first of 10 repeated scanning blocks, each of which consisted of a counter‐balanced ordering of a resting‐state BOLD (6 min), ASL (5 min), and finally a Field Mapping sequence (acquired for quality control, 1 min). Throughout the scan, participants were asked to fixate on a projected cross and “think about nothing in particular” but remain awake. A “Baseline” block was followed by a “Stimulation” block, during which scans were acquired while tDCS was turned on and delivered for 15 min, as previously described. At the end of the stimulation, the tDCS device was turned off and the final eight resting state blocks were acquired in intervals of 15 min until 120 min after the end of stimulation (Figure 1c). At the end of each block (~12 min), the participant was instructed to use a response pad to rate their tiredness/arousal level on a visual analog scale, where the lowest value “0” denoted “not tired at all” and the highest value “10” indicated “extremely tired.” Note that even though the respective analysis revealed no changes in tiredness throughout the session (mean score = 4.02, no significant effect of time (*p* = .93) or session (*p* = .688)), this questionnaire task was mainly used to ensure participants remained attentive and awake throughout the blocks.

### 2.6 Data analyses and statistics

#### 2.6.1 TMS experiment

All details regarding the processing and statistical analysis of the MEP data are presented in the former study (Jamil et al., 2017). In order to confirm that the subgroup of participants included in the present study accord with the findings of the larger study, the statistical analyses were conducted again on the present cohort. Briefly, the peak‐to‐peak amplitudes of the 25 MEPs for each time epoch were calculated and pooled together per timepoint. The distribution of the data was assessed using the Kolmogorov–Smirnov procedure, and no significant deviations from normality were detected. To determine if individual baseline measures differed between sessions, we entered SI_1mV_ and Baseline MEP as dependent variables in a repeated measures ANOVA with intensity as a within‐subject factor. As this ANOVA did not reveal a significant main effect for session during the baseline (Table 1), all MEP amplitudes were normalized to the pre‐intervention baseline to obtain values representing the subject‐ and session‐specific relative change in excitability in the following manner:

$$\mathrm{∆MEP}=\frac{{\mathrm{MEP}}_{t}-{\mathrm{MEP}}_{\text{Baseline}}}{{\mathrm{MEP}}_{\text{Baseline}}}$$

**Table 1:** Baseline measurements in global cerebral blood flow (CBF) and baseline amplitude of the motor‐evoked potential (MEP) (±*SD*). CBF was quantified from perfusion images and calibrated against a reference proton density image using a single compartment model as previously described (see Section 2). No factor differed significantly between session and experimental group

Experiment	Experimental session	Global mean baseline CBF (ml/100 mg/min)	Baseline MEP (mV)
Anodal stimulation	Sham	58.41 ± 5.00	0.98 ± 0.15
	0.5 mA	56.82 ± 3.50	0.92 ± 0.15
	1.0 mA	58.97 ± 3.59	0.97 ± 0.17
	1.5 mA	59.38 ± 4.05	0.91 ± 0.15
	2.0 mA	58.01 ± 2.55	0.97 ± 0.21
Cathodal stimulation	Sham	60.82 ± 5.98	0.92 ± 0.13
	0.5 mA	60.24 ± 5.57	0.96 ± 0.09
	1.0 mA	59.47 ± 5.75	0.93 ± 0.11
	1.5 mA	57.78 ± 5.40	0.98 ± 0.10
	2.0 mA	59.18 ± 5.37	0.97 ± 0.16

This resulted in relative values representing either increased or decreased excitability. The normalized MEPs were entered as dependent variables into a 2‐way repeated measures ANOVA, with within‐subject factors intensity (5 levels) and time (10 levels). Mauchly's test of sphericity was conducted, and Greenhouse–Geisser correction was applied when necessary. As time‐series data were normalized with respect to baseline, we conducted a priori one‐sample *t*‐tests at each poststimulation timepoint in order to assess respective changes compared to baseline (“0”). In the case of significant main effects or interactions, further exploratory follow‐up tests were conducted using Student's paired *t*‐test. All *t*‐tests were two‐tailed, with alpha level set to *p* < .05, and not corrected for multiple comparisons.

#### 2.6.2 fMRI preprocessing

Figure 1d presents an overview of the preprocessing pipeline. ASL image preprocessing steps were carried out using the freely available FSL package, version 5 (http://fmrib.ox.ac.uk/fsl). The first four pcASL volumes were discarded to allow for magnetization equilibrium, and remaining volumes were slice‐time corrected. Motion correction was conducted in two steps in order to address low and high levels of head motion (Ciric et al., 2017). In a first step to correct micromovements, all volumes within a run were realigned to the first volume in the timeseries, and six estimates of motion (*x*, *y*, *z*, pitch, yaw, and roll) as well as their first derivatives were extracted, using the command MCFLIRT in FSL v5 (Jenkinson, Bannister, Brady, & Smith, 2002). These parameters were taken to a second step, where larger deviations of head movement were corrected using a censoring method based on a criterion of the framewise displacement greater than 0.5 mm between sequential volumes (Ciric et al., 2017). In cases matching this criterion, the preceding or subsequent volume was also censored, depending on whether the volume was a “tag” or “control” ASL scan. Perfusion‐weighted images were calculated by a pair‐wise subtraction of tag and control volumes, which were then input into a one‐compartment kinetic model describing blood transit based on labeling and postlabel delay times (using the model parameters based on (Alsop et al., 2015)), and further calibrated into absolute CBF values using the acquired PD image as per (Chappell, Groves, Whitcher, & Woolrich, 2009). Image volumes were spatially normalized in a two‐step procedure: first, coregistration to the subject's high resolution T1‐weighted anatomical image using boundary‐based registration (Greve & Fischl, 2009), and then realignment to the Montreal Neurological Institute (MNI) standard 2 mm brain image by means of FSL's linear registration tool, FLIRT (Jenkinson et al., 2002). Lastly, images were spatially smoothed using an 8 mm full‐width at half‐maximum (FWHM) Gaussian kernel.

#### 2.6.3 fMRI statistical analysis

**Figure 2:**
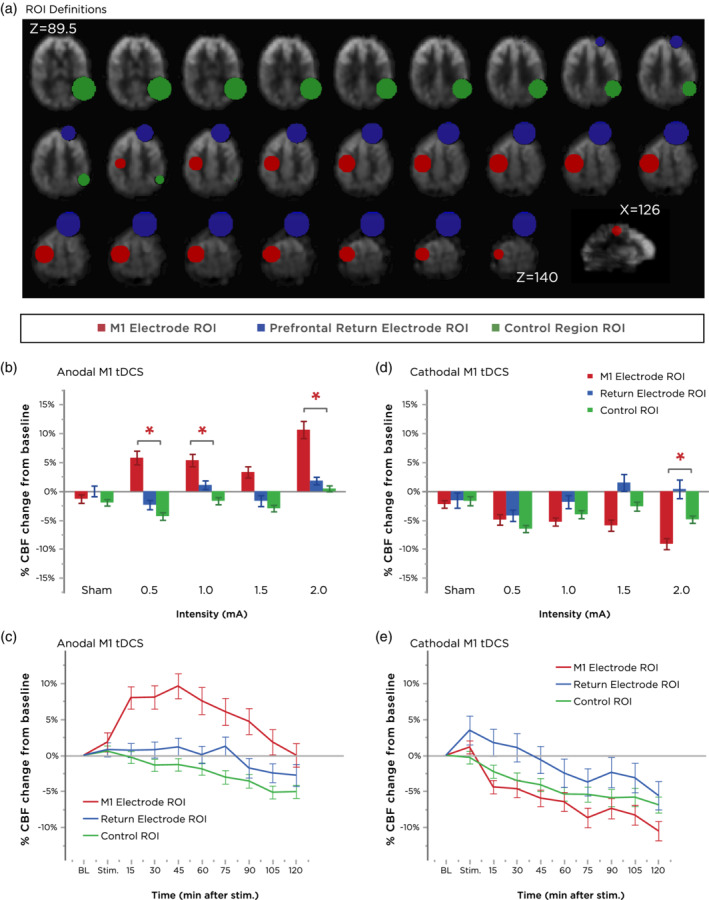
Regional modulation of cerebral blood flow (CBF). (a) Axial slices extracted from a representative subject's mean perfusion‐weighted functional scan are labeled with definitions of the ROI masks used in the main analysis. Error bars represent the SEM. (b,c) Summary of the effects of different anodal M1 tDCS intensities (grand‐averaged over all timepoints in (b) and over all intensities in (c)) on each ROI. Note the selective effect of all intensities on CBF modulation in the target electrode/left hand M1 hand knob ROI, which persisted for the entire scanning duration. Error bars represent the SEM. (d,e) Summary of the effects of different cathodal M1 tDCS intensities (grand‐averaged over all timepoints in (d) and over all intensities in (e)) on each ROI. With the exception of 0.5 mA, the greatest decrease in CBF was again observed in the target electrode ROI. Cathodal‐M1 tDCS over all intensities induced a decrease in perfusion in the left M1 region, but a slight increase in perfusion in the right prefrontal region (location of the anodal electrode). Error bars represent the SEM. ROI, regions of interest; tDCS, transcranial direct current stimulation

A first analysis was conducted to quantitatively assess changes in cerebral perfusion directly under the tDCS electrodes. As such, we extracted perfusion time courses between three regions of interest (ROIs) (Figure 2a visualizes these ROIs on axial cross‐sections):

1. The region of the target electrode, the hand knob motor representation area, which was defined in the cortex using a 1.5 cm radius sphere centered at MNI coordinates (*x* = −37.4, *y* = −19.1, *z* = 52.4 mm). These coordinates were obtained from an ASL/BOLD study which functionally localized the hand motor area with respect to local vasculature, and with high inter‐subject agreement (Pimentel, Vilela, Sousa, & Figueiredo, 2013).
2. The region of the return electrode, the right frontal orbit and anterior sections of the prefrontal cortex, which was defined as a 5 cm radius sphere centered in the midpoint of the orbital regions of the superior frontal gyri, inferior to the anterior commissure/posterior commissure plane, and the inferior portion of the right prefrontal cortex (MNI coordinates: *x* = 23.5, *y* = 50, *z* = 16.5 mm).
3. A control region, in order to rule out that changes in perfusion were driven by nonspecific effects altering global perfusion. The control region was chosen in order to be anatomically and functionally remote from, and of the same volume as the target electrode ROI (Stagg, O'Shea, et al., 2009; Zheng et al., 2011). This ROI was defined as a 1.5 cm sphere centered in the right superior temporal gyrus (temporo‐occipital ROI, MNI: *x* = 58, *y* = −58, *z* = 10 mm).

For each individual session, the grand‐average mean perfusion time course of the voxels in each ROI from the 4‐dimensional volume was extracted using routine functions and then averaged over the time‐series, resulting in 10 mean perfusion values per session that were assessed in a statistical model. A first ANOVA to assess whether CBF at baseline differed between sessions for each ROI did not reveal a significant main effect for session (Table 1); thus, perfusion values were normalized to the prestimulation baseline to obtain values representing the subject‐ and session‐specific relative change in perfusion in the following manner:

$$∆r\mathrm{CBF}=\frac{{r\mathrm{CBF}}_{t}-{r\mathrm{CBF}}_{\text{Baseline}}}{{r\mathrm{CBF}}_{\text{Baseline}}}$$

As such, positive values represented a postintervention increase in perfusion whereas negative values represented a decrease. To determine whether poststimulation CBF changed with respect to baseline, we conducted a priori one‐sample *t*‐tests at each poststimulation timepoint in order to assess respective changes compared to baseline (0). Inspection of normality by means of Kolmogorov–Smirnov tests did not indicate any major outliers or deviations from normality at the group level, and the between‐subject factor met the criteria of equality of variance as assessed by the Levene and Brown–Forsythe test (all values of *p* > .05); thus, standard parametric procedures were followed. Polarity, time, and intensity‐dependent effects were assessed by means of a mixed‐design repeated‐measure ANOVA (3 levels of factor ROI, 5 levels for factor intensity, 9 levels for factor time, and between‐subject factor polarity). Violations of nonsphericity, indicated by Mauchly's test, were addressed using the Greenhouse–Geisser correction when necessary. Estimates of effect size are presented with the partial‐ETA‐squared (*ηp* ^2^) metric. In the case of a significant main effect of intensity, we conducted exploratory *post‐hoc* comparisons (two‐tailed paired *t*‐tests) between the respective tDCS intensity and sham conditions. In the case of a significant main effect or interaction effect of time, we performed exploratory *post‐hoc* comparisons over a reduced dataset by first averaging timepoints 0–60 and 60–120 min of the time‐series, which was a reasonable trade‐off to reduce both Type I and Type II error (i.e., reduce the number of multiple comparisons while remaining sensitive to any differences across the temporal scale, as seen from the time‐course of MEP changes). Respective differences were assessed by two‐tailed paired *t*‐tests. As these follow‐up tests were exploratory, *p*‐values were not adjusted for multiple comparisons.

### 2.7 Correlation analyses

#### 2.7.1 Correlation between motor cortical excitability and cerebral perfusion

To compare intensity‐ and polarity‐wise effects of tDCS on cortical excitability with changes in perfusion, we performed a *post‐hoc* correlation analysis. The analysis was performed at the individual level, using each subject's mean change from baseline over two 60 min intervals with respective changes in CBF. We performed the analysis separately with the three aforementioned ROIs in the first analysis (the target electrode/left M1 hand knob region, the return electrode/right prefrontal region, and a final control/right temporo‐occipital region). In addition, we included also a larger regional network ROI which contained the left sensorimotor network (Brodmann areas corresponding to the somatosensory, primary motor, and premotor cortex). This additional ROI was included, based on the evidence that TMS‐MEP are influenced by inter‐regional activity stemming from afferent inputs into the motor cortex from the ipsilaterally connected premotor and somatosensory cortices, thereby contributing to activation of the direct cortico‐spinal projections onto spinal motor neurons (Bestmann & Krakauer, 2015; Reis et al., 2008). As such, and on the basis of our previous tDCS‐fMRI functional connectivity study (Polanía, Paulus, & Nitsche, 2012), we hypothesized that tDCS may have influenced the functional architecture of intra‐regional sensorimotor circuits, spanning different populations of M1 neurons, and thereby leading to the observed changes in the motor‐evoked potential. For each of the four active intensities, we averaged each individual's mean baseline‐normalized change in perfusion within these four ROIs over the two 60‐min time intervals, subtracted them from the corresponding sham data, and compared them with respective baseline‐normalized changes in excitability. We standardized the two sets of data by means of Fisher's *z*‐transformation and then performed correlation calculations using Pearson's method.

#### 2.7.2 Correlation to electric field model

In order to assess whether CBF activations across the cortex obtained using fMRI agree respectively with computationally modeled electric field strengths, we generated a realistic finite element model of our electrode montage on the MNI template head using SimNIBS v2.1.2 (Thielscher, Antunes, & Saturnino, 2015). The target electrode was positioned over the hand‐knob region extrapolated to the surface of the MNI head and rotated at an angle of 45° (*x* = −50.57, *y* = −22.84, *z* = 80.32). The return electrode was positioned over the contralateral supraorbital ridge, with the center point approximately over the AF4 EEG position (*x* = 47.66, *y* = 66.69, *z* = 19.75). Conductivity constants for tissue compartments were set to default values (WM: 0.126 S/m, GM: 0.275 S/m, CSF: 1.654 S/m; bone: 0.010 S/m, compact bone: 0.008 S/m, and spongy bone: 0.025 S/m). Additional parameters were set to account for the correct positioning of the electrode connector positions as well as the conductivity medium of the gel (Saturnino et al., 2015). The resulting model was visualized using Gmesh, and the norm of the electric field matrix was converted to a NIFTI volume in MNI space. Finally, voxelwise rank correlations using Spearman's correlation between the predicted electric field strength and effective changes in CBF were calculated using the group‐level T‐contrast images for each active tDCS intensity obtained from a whole‐brain analysis (see Supporting Information 1).

## 3 RESULTS

### 3.1 No baseline differences between motor thresholds, MEP, or CBF

Table 1 summarizes the descriptive statistics of the study sample at hand. At baseline, between‐session differences in baseline motor cortical excitability did not significantly differ for both the anodal (*F*[4,56] = 1.06, *p* = .385) or the cathodal group (*F*[4,52] = 0.351, *p* = .842). Note that as the TMS SI_1mV_ intensity was adjusted for each subject at baseline to reach a stable MEP, we would not expect to find significant effects here as long as baseline excitabilities were stable. We also assessed differences in individual TMS SI_1mV_ intensities and found no significant differences for either group (anodal: *F*(4,56) = 1.881, *p* = .126; cathodal: *F*(4,52) = 0.833, *p* = .510), indicating inter‐session variability in baseline motor thresholds was not a relevant factor. Similarly, for the ASL‐fMRI data, global CBF did not significantly differ at baseline for either group of anodal (*F*(4,56) = 1.444, *p* = .232) or cathodal tDCS (*F*(4,52) = .642, *p* = .637). Moreover, within‐subject baseline CBF was fairly reliable across sessions (intra‐class correlation coefficient ICC[2,1] = 0.590, *n* = 29; Cronbach's alpha = 0.878, *n* = 5).

### 3.2 Polarity and intensity‐dependent modulation of CBF

Quantitative changes in CBF at the five intensities across nine measurement time‐points were assessed using ANOVAs. An overall mixed‐model ANOVA with factors ROI, polarity, intensity, and time, indicated significant main effects of ROI (*F*[2,52] = 12.053, *p* = <.001, *ηp* ^2^ = 0.317), polarity (*F*[1,26] = 6.210, *p* = .019, *ηp* ^2^ = 0.193), and time (*F*[4.136,107.529] = 17.295, *p* = <.001, *ηp* ^2^ = .399) as well as significant interactions between ROI × polarity (*F*[2,52] = 32.790, *p* = <.001, *ηp* ^2^ = 0.558), polarity × time (*F*[4.136,107.529] = 5.068, *p* = .001, *ηp* ^2^ = 0.163), ROI × polarity × intensity (*F*[4.940,128.448] = 2.345, *p* = .046, *ηp* ^2^ = 0.083), and ROI × polarity × time (*F*[7.573,196.900] = 3.826, *p* = <.001, *ηp* ^2^ = 0.128). Inspection of the ROI × polarity × intensity interaction indicated that anodal‐M1 tDCS with all active intensities induced the larger effect in the targeted left M1 hand knob area compared with the control region (Figure 2b). For cathodal‐M1 tDCS, the same pattern held, but for reduction in CBF, with the exception of the 0.5 mA intensity (Figure 2d). As seen in the plot of the ROI × polarity × time interaction, anodal‐M1 tDCS resulted in more increased CBF in the M1 hand knob region relative to the other ROIs, which was maximum 45 min after stimulation before returning toward baseline, while no changes were observed in the return electrode region, and a slight decreasing drift in CBF in the control region (Figure 2c). For cathodal‐M1 tDCS, CBF under the M1 electrode decreased the greatest relative to the other ROIs, which was largest 120 min after stimulation. An initial enhancement in CBF was observed in the return electrode region, which then returned and drifted below baseline, similar to the control ROI (Figure 2e). In order to further dissect the interactions in the overall ANOVA, we re‐performed two‐way ANOVAs (intensity × time) for each ROI and polarity combination.

#### 3.2.1 Anodal‐M1 tDCS

**Figure 3:**
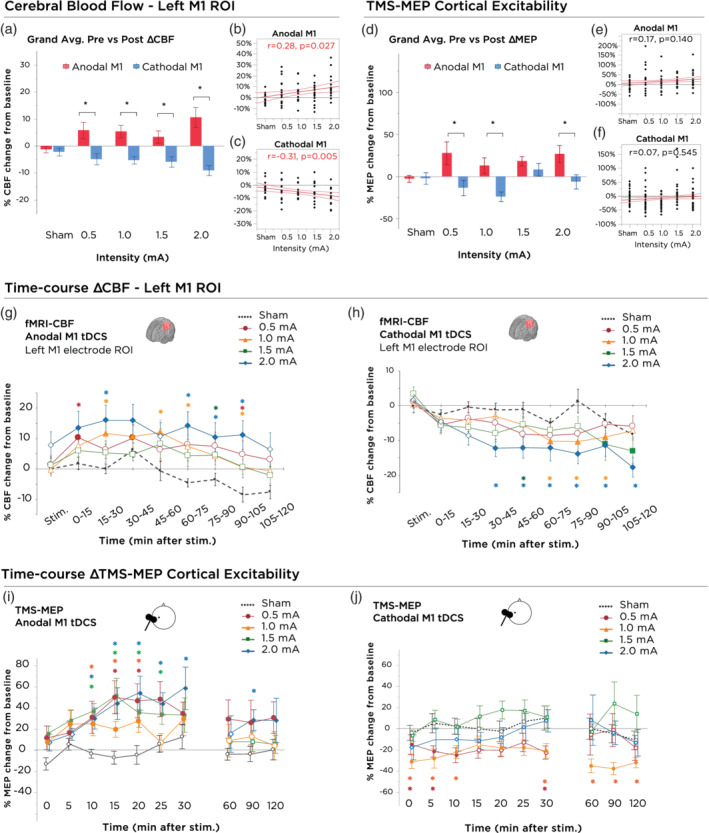
Stimulation intensity and polarity dependent effects of tDCS on motor cortex excitability and local cerebral blood flow. (a) 0–120 min grand‐averaged after‐effects of cerebral blood flow following 15 min of anodal and cathodal stimulation at intensities ranging from sham‐2.0 mA within the target electrode ROI (left M1 hand knob region). Asterisks indicate significant differences between polarities (unpaired *t*‐test, *p* < .05). Polarity‐dependent differences were significant for all active tDCS intensities. Error bars indicate the SEM. (b) Correlation between current intensity and grand‐average change in CBF following anodal‐M1 tDCS. Red lines indicate the 95% CI. (c) Correlation between current intensity and grand‐average change in CBF following cathodal‐M1 tDCS. Red lines indicate the 95% CI. (d) 0–120 min grand‐averaged after‐effects of cortical excitability following 15 min of anodal and cathodal stimulation at intensities ranging from sham‐2.0 mA on the mean MEP amplitude. Asterisks indicate significant differences between polarities (unpaired *t*‐test, *p* < .05). Polarity differences were significant with current intensities of 0.5, 1.0, and 2.0 mA. Error bars indicate the SEM. (e) Correlation between current intensity and grand‐average change in motor cortex excitability following anodal‐M1 tDCS. Red lines indicate the 95% CI. (f) Correlation between current intensity and grand‐average change in motor cortex excitability following cathodal‐M1 tDCS. Red lines indicate the 95% CI. (g) Time‐course changes of CBF within the left M1 hand knob ROI following anodal M1 tDCS. 2.0 mA resulted in significantly elevated CBF, compared to sham, which persisted over the majority of the 2 hr session and peaked between 30–45 min after tDCS. Error bars indicate the SEM. (h) Time‐course changes of CBF within the left M1 hand knob ROI following cathodal M1 tDCS. 2.0 mA resulted in significantly decreased CBF, compared to sham as well as baseline, which lasted the entire 2 hr session. Delayed onset after‐effects were observed for the 1.0 mA intensity, between timepoints 60–105 min. Other intensities, although not significant, led to trendwise identically directed effects. Error bars indicate the SEM. (i) Time‐course changes in cortical excitability following anodal‐M1 tDCS. Filled symbols indicate a significant difference in cortical excitability against the “0” baseline (one‐sample *t*‐test, two‐tailed, *p* < .05). Floating symbols indicate a significant difference between the active intensity and sham stimulation (paired *t*‐test, two‐tailed, *p* < .05). Anodal stimulation over all active intensities resulted in significant increases of excitability lasting up to 30 min. Sham stimulation did not induce any significant change in cortical excitability. Error bars indicate the SEM. (j) After‐effects of cortical excitability following 15 min of cathodal stimulation at intensities ranging from sham‐2.0 mA on the mean MEP amplitude. Filled symbols indicate a significant difference in cortical excitability against the “0” baseline (one‐sample *t*‐test, two‐tailed, *p* < .05). Floating asterisks indicate a significant difference between the active intensity and sham stimulation (paired *t*‐test, two‐tailed, *p* < .05). Only 0.5 and 1.0 mA cathodal stimulation resulted in significant differences from baseline, and only 1.0 mA was significantly different from sham through the later time bins. Higher intensities such as 1.5 and 2.0 mA tended to return to baseline values after about 10 min. Sham stimulation did not induce any significant change in cortical excitability. Error bars indicate the SEM. MEP, motor evoked potential; ROI, regions of interest; tDCS, transcranial direct current stimulation

**Figure 4:**
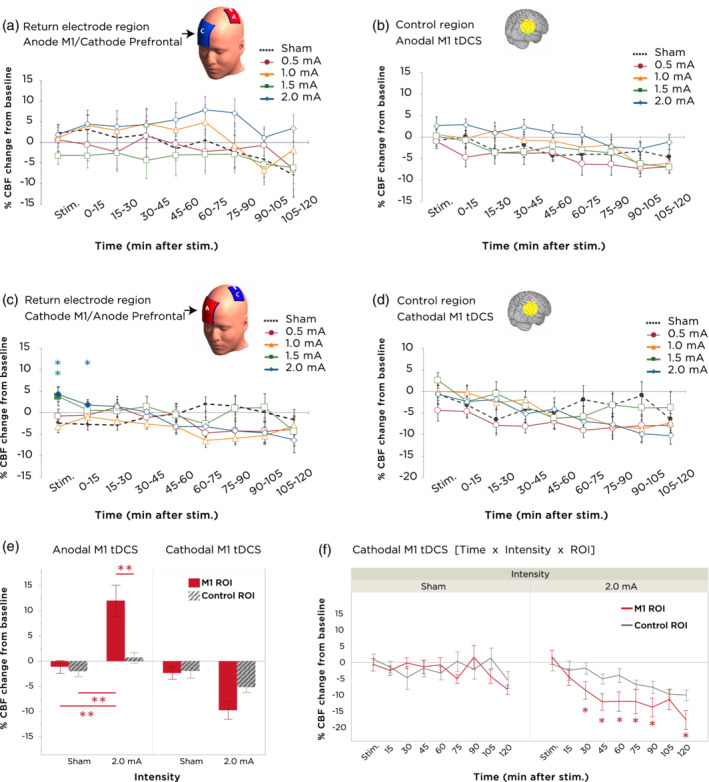
Cerebral blood flow alterations at return electrode and control ROIs. (a) Time‐course changes of CBF within the return electrode ROI following anodal M1 tDCS. No significant differences between intensities or timepoints were obtained. Error bars indicate the SEM. (b) Time‐course changes of CBF within the control region ROI following anodal M1 tDCS. No significant intensity or timepoint differences were found. Error bars indicate the SEM. (c) Time‐course changes of CBF within the return electrode ROI following cathodal M1 tDCS. An increase in CBF compared to sham was observed with 1.5 mA and 2.0 mA during the stimulation block. Error bars indicate the SEM. (d) Time‐course changes of CBF within the control region ROI following cathodal M1 tDCS. No significant differences in stimulation intensity or timepoint were found. Error bars indicate the SEM. (e) Evaluation of the anatomical specificity of anodal‐ and cathodal‐M1 tDCS through an interaction analysis between (1) changes in CBF between sham tDCS and 2.0 mA tDCS with (2) changes in CBF between the target M1 ROI vs. the control ROI. For anodal‐M1 tDCS, a significant ROI × intensity interaction indicates greater increase in CBF with 2.0 mA at the target M1 site, but not in the control ROI, when compared to sham tDCS. Double asterisks indicate values of *p* < .001 (paired *t*‐tests), and error bars indicate the SEM. (f) For cathodal‐M1 tDCS, a time × intensity × ROI interaction is observed, which indicates decreased CBF in the target M1 region relative to the control ROI during the timepoints between 30–90 and 120 min after 2.0 mA tDCS. Asterisks indicate values of *p* < .05 (paired *t*‐tests), and error bars indicate the SEM. CBF, cerebral blood flow; ROI, regions of interest; tDCS, transcranial direct current stimulation

For the ROI of the left M1 hand knob region (region directly under the left M1 target electrode), the ANOVA indicated significant main effects of intensity (*F*(4,56) = 2.644, *p* = .043, *ηp* ^2^ = 0.159) and time (*F*[3.679,51.501] = 10.351, *p* < .001, *ηp* ^2^ = 0.425). No main effect of intensity or an intensity × time interaction was observed for other ROIs. Furthermore, the ANOVA revealed a significant linear relationship between (increased) CBF as a function of (increased) current intensity (*F*[1,14] = 6.086, *r* = 0.28, *p* = .027). While 0.5 mA tDCS showed weak effects overall when compared to other intensities, an initial enhancement of CBF was observed within during first 15 min after stimulation when compared to sham (Figure 3g). 1.0 mA tDCS induced an increase in CBF significantly greater than sham after 15 min following tDCS, which persisted up to 105 min following the end of stimulation. 1.5 mA tDCS also resulted in increased CBF in the target left M1 site, with significant differences compared to sham detected after a delay of 75 min after the end of stimulation. More profoundly, the effects of 2.0 mA were the strongest among all intensities: CBF increased significantly from 15 min following the end of stimulation and remained elevated up to 105 min afterward, with the maximum CBF increase of 16.1% from baseline (15.3% relative to sham) after 30 min. In the ROI of the return cathodal electrode (right prefrontal spherical area), the ANOVA showed a main effect of time (*F*[3.143,44.001] = 2.859, *p* = .045, *ηp* ^2^ = 0.170), but no main effect of intensity or intensity × time interaction. As seen in Figure 4a, CBF in this region was observed to slowly decrease throughout the time‐course of the session. To assess whether this time‐course drift in CBF was nonspecific (i.e., as a result of systematic changes in arousal and not specifically due to tDCS), we performed an additional analysis to assess changes in CBF in a remote ROI area (see Section 2). The ANOVA for this control region also revealed a main effect of time (*F*[4.107,57.503] = 16.078, *p* = <.001, *ηp* ^2^ = 0.535), but no main effects of intensity or intensity × time interaction. Thus, all ROIs showed a main effect of time, including the control ROI, which was characterized by a slow and gradual decrease in CBF in the range of 0–5% over the 2 hr of scanning (Figure 4b).

#### 3.2.2 Cathodal‐M1 tDCS

An ANOVA of the mean CBF differences in the targeted left motor hand knob area showed a marginally nonsignificant main effect of intensity (*F*(4,52) = 2.415, *p* = .060, *ηp* ^2^ = .157), a significant effect of time (*F*[4.320,56.164] = 12.944, *p* = <.001, *ηp* ^2^ = 0.499) and a significant intensity × time interaction (*F*[32,416] = 1.943, *p* = .002, *ηp* ^2^ = 0.130). In addition, the ANOVA revealed a linear (negative) relationship between intensity and CBF (*F*[1,13] = 11.579, *r* = −0.31, *p* = .005). For the return electrode ROI, a significant intensity × time interaction was shown (*F*[7.771,101.028] = 2.693, *p* = .011, *ηp* ^2^ = 0.172). No effect of intensity or the intensity × time interaction was observed for the control ROI (Figure 4d). In follow‐up *post‐hoc* tests, all active intensities showed a trend of decreased CBF in the targeted left M1 area (Figure 3h). No significant differences were observed with 0.5 mA. For 1.0 mA, CBF decreased significantly relative to sham from 60 min post‐tDCS until up to 105 min. 1.5 mA cathodal tDCS induced a CBF decrease which was significant only at timepoint 45–60 min poststimulation. 2.0 mA cathodal tDCS resulted in the largest overall decrease, beginning 30 min after stimulation and persisting until the end of the monitoring (all values of *p* < .05; 17.7% decrease relative to baseline, 9.3% decrease relative to sham at 120 min). Interestingly, when assessing the time‐course changes of CBF in the anodal return electrode region, we observed that higher intensities of 1.5 and 2.0 mA increased CBF during the stimulation period before declining back toward baseline over the remainder of the session (Figure 4c).

#### 3.2.3 Anatomically specific effects of 2.0 mA tDCS

Given that the highest tested intensity (2.0 mA) yielded the greatest changes in blood flow for both anodal‐ and cathodal‐M1 tDCS, we asked whether the spatial distribution of perfusion changes with these highest intensities remained anatomically specific to the targeted M1 area. We therefore performed separate *post‐hoc* rm‐ANOVAs for each anodal‐ and cathodal‐M1 tDCS, with main factors being intensity (2 levels: sham and 2.0 mA), ROI (2 levels: M1 and control region), and time (9 levels). For anodal‐M1 tDCS, we observed significant main effects of time (*F*[8,112] = 5.789, *p* < .001), intensity (*F*[1,14] = 7.339, *p* = .017), ROI (*F*[1,14] = 0.008), and a significant ROI × intensity interaction (*F*[1,14] = 7.248, *p* = .018). This latter interaction (Figure 4e) indicated that 2 mA anodal‐M1 tDCS, but not sham, increased CBF in the M1 ROI relative to the control ROI (*p* < .001, Student's paired *t*‐test). For cathodal‐M1 tDCS, we found significant main effects of time (*F*[8,104] = 7.529, *p* < .001), intensity (*F*[1,13] = 11.781, *p* = .005), and ROI (*F*[1,13] = 5.6586, *p* = .033). Here, although the intensity × ROI interaction was marginally nonsignificant (*F*[1,13] = 4.318, *p* = .058), we did observe a significant three‐way interaction between time × intensity × ROI (*F*[8,104] = 3.528, *p* = .0012). A follow‐up test of this interaction comparing the effects of each intensity between the two ROIs (Figure 4f) showed that while there were no significant differences in CBF changes with sham tDCS between the two ROIs, for 2.0 mA cathodal‐M1 tDCS, CBF decreased in the M1 region relative to the control ROI only beginning from 30 min after stimulation, and which remained decreased for nearly the rest of the scanning duration with the exception of the 105‐min poststimulation timepoint (all values of *p* < .05, Student's paired *t*‐test).

### 3.3 Polarity and intensity‐dependent effects of tDCS on TMS motor‐cortical excitability

Excitability changes were statistically assessed by means of repeated measures ANOVAs. An overall ANOVA with polarity as a between subject factor revealed a significant effect of polarity (*F*[1,27] = 23.093, *p* < .001, *ηp* ^2^ = 0.461), intensity (*F*[2.890,78.040] = 3.214, *p* = .029, *ηp* ^2^ = 0.106), time (*F*[3.973,107.274] = 4.442, *p* = .002, *ηp* ^2^ = 0.141), and a significant polarity × intensity interaction (*F*[2.890,78.040] = 4.094, *p* = .010, *ηp* ^2^ = 0.132). The polarity × intensity interaction was statistically evaluated between the two polarities at each intensity using unpaired *t*‐tests, as presented in Figure 3d. With exception of 1.5 mA, cortical excitability was significantly enhanced after anodal compared to cathodal tDCS for each active intensity. We also assessed whether the intensity‐dependent effects of each polarity scaled in a linear manner by assessing the fit of the first‐order polynomial degree, and we found no significant linear effect for either anodal or cathodal tDCS (Figure 3e,f, *p* > .05). To dissect the polarity‐dependent effect of each intensity further, ANOVAs were conducted for each polarity separately and we summarize the main findings as follows:

#### 3.3.1 Anodal‐M1 tDCS

A two‐way (intensity × time) rm‐ANOVA indicated a significant main effect of intensity (*F*(4,56) = 3.044, *p* = .024, *ηp* ^2^ = 0.179), and time (*F*[3.783,52.960] = 4.699, *p* = .003, *ηp* ^2^ = 0.251), but no intensity × time interaction (*F*[7.681,107.538] = 1.038, *p* = .411, *ηp* ^2^ = 0.069). No linear association was observed for the factor intensity (Figure 3e). Intensity and time‐course changes are plotted in Figure 3i, where timepoints showing significant differences between baseline and post‐tDCS measurements were assessed by a priori one‐sample *t*‐tests, and active tDCS intensities showing significant differences versus sham assessed by paired sample *t*‐tests. Comparisons of active tDCS intensities to sham revealed that all active intensities resulted in a significant poststimulation increase in cortical excitability (Figure 3i). Sham stimulation did not result in a change of cortical excitability. With regard to temporal effects, although no discernable differences were detected in the first 30‐min after stimulation, both 2.0 and 0.5 mA remained at elevated excitability up to 120 min after stimulation relative to baseline, whereas other active intensities showed a tendency to return to baseline excitability levels.

#### 3.3.2 Cathodal‐M1 tDCS

The two‐way (intensity × time) rm‐ANOVA indicated only an effect of intensity (*F*(4,52) = 4.882, *p* = .008, *ηp* ^2^ = .273), but no effect of time (*F*[2.704,35.154] = 1.039, *p* = 0.382, *ηp* ^2^ = 0.074) or intensity × time interaction (*F*[5.363,69.721] = 0.921, *p* = .478, *ηp* ^2^ = 0.066). No linear association between current intensity and excitability changes were observed (Figure 3f). As seen in the time‐course plots of poststimulation changes in excitability (Figure 3j), the lower intensities of 0.5 and 1.0 mA resulted in excitability diminution relative to both baseline and sham conditions, with 0.5 mA persisting up to 30 min and 1.0 mA persisting up to 120 min. Interestingly, we found that the magnitude of the effects of 1.0 mA cathodal tDCS relative to sham was greater in the later epoch (60–120 min) compared to earlier timepoints. 1.5 and 2.0 mA intensities did not show significant differences compared to baseline values (Figure 3j). Sham stimulation also resulted in no effect.

### 3.4 Correlation between tDCS‐induced modulation of cortical excitability (TMS‐MEP) and cerebral perfusion (fMRI‐CBF)

**Figure 5:**
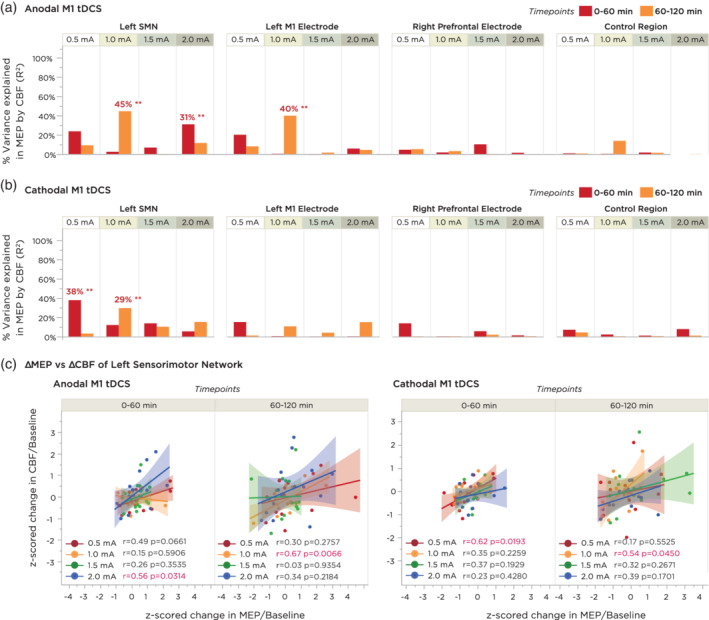
Correlation analyses between time‐binned averages in motor cortical excitability and cerebral blood flow. MEPs were compared to CBF values from four regions of interest: (I) the left sensorimotor network consisting of the somatosensory, primary and premotor cortices; (II) the anatomical region underneath the target electrode, corresponding to the left M1 hand knob area; (III) the anatomical region underneath the return electrode, corresponding to the right prefrontal area; and (IV) an ROI of the same size as the M1 ROI located over the right temporo‐occipital area. Correlations were calculated using linear/Pearson correlation between each subject's *z*‐normalized change in CBF versus their *z*‐normalized change in MEP, averaged across two 60‐min time bins. (a) Summary of percentage variance explained (*R* ^2^ value) for each of the above comparisons for anodal M1 tDCS. Double asterisks indicate a significant correlation (*p* < .05). (b) Summary of percentage variance explained for each of the above comparisons for cathodal M1 tDCS. Note that both anodal and cathodal M1 tDCS showed stronger associations between MEP and CBF changes within the Left SMN ROI. (c) Expanded plots of the correlation between change in CBF and MEP within the left SMN ROI for both anodal and cathodal M1 tDCS. Shaded background regions indicate the 95% CI. CBF, cerebral blood flow; MEP, motor evoked potential; ROI, regions of interest; tDCS, transcranial direct current stimulation

In order to quantitatively assess whether intensity and polarity‐dependent changes in motor cortex excitability correlated with individual changes in cerebral perfusion, we calculated correlation coefficients between baseline‐normalized changes in motor cortical excitability with changes in CBF. Excitability changes were compared with four CBF ROIs: the three spherical ROIs corresponding to the regions of the M1 target electrode, the prefrontal return electrode, and the control ROI, as well as a final ROI containing the left primary and premotor network (Brodmann regions 1, 4, and 6; see Section 2). Figure 5 presents a summary of the results (complete correlation plots can be found in Supporting Information 2).

#### 3.4.1 Anodal‐M1 tDCS

Individual‐level changes in cortical excitability and sensorimotor perfusion showed the strongest association with the 1.0 mA intensity during the 60–120 min time interval (*r* = 0.667, *p*_FDR_ = 0.023). We also observed a tendency for 0.5 mA and 2.0 mA to correlate positively in the first 0–60 min, although these correlations did not reach significance after FDR adjustment for multiple comparisons (*r* = 0.486, *p*_FDR_ = 0.188, and *r* = 0.556, *p*_FDR_ = 0.126, respectively). For the left M1 hand knob ROI, the strongest association was again observed with 1.0 mA (*r* = 0.631, *p*_FDR_ = 0.023), which was however not stronger than the correlation observed in the sensorimotor region. Positive trend‐wise associations between increased CBF as a function of increased excitability were also observed with the 0.5 and 2.0 mA intensities in this ROI, although these correlations did not reach statistical significance. For the right prefrontal ROI and the right temporo‐occipital ROI, no significant correlations or otherwise trends were observed at any intensity (Figure 5a).

#### 3.4.2 Cathodal‐M1 tDCS

For the sensorimotor region ROI, no significant correlation was observed after FDR correction for multiple comparisons; however, a trend‐wise positive correlation between poststimulation change in MEP and poststimulation change in CBF was observed with 0.5 mA during the first 60 minutes only (*r* = 0.615, *p*_FDR_ = 0.077) and with 1.0 mA during the second 60 min (*r* = 0.542, *p*_FDR_ = 0.180). For the left M1 hand knob ROI, right prefrontal ROI, and the right temporo‐occipital ROI, no significant correlations were detected (Figure 5b).

### 3.5 Correlation between predicted electric field strength and cerebral perfusion

**Figure 6:**
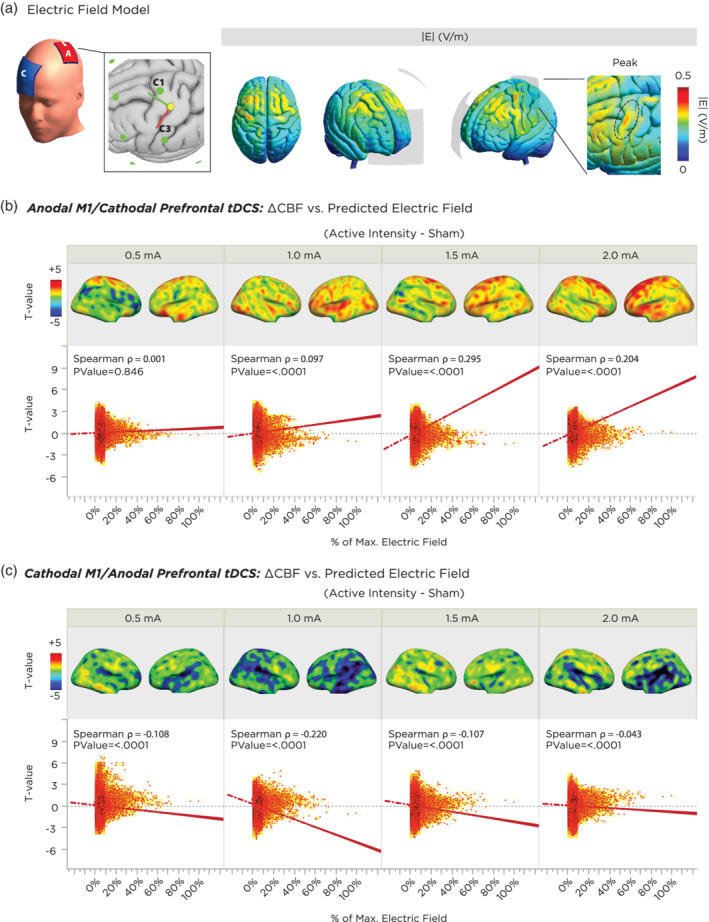
Correlation between a realistic finite element model of the predicted changes in the electric field of cortical gray matter and actual physiological findings from the experiment. (a) Location of target and return electrodes on the MNI template head. The return electrode was positioned over position the “AF4” EEG position, and the M1 electrode was placed according to MNI‐standardized coordinates of the hand knob region. The resulting montage was segmented using the finite‐element‐method across anatomical tissue layers, and the electric field was computed, which showed a maximum peak underneath, and along the anterior edge of the target electrode. (b) Correlation between predicted electric field strength and tDCS‐induced CBF changes as a function of current intensity for anodal‐M1 tDCS. The top panels summarize the grand‐average *T‐*contrast between the active tDCS intensity vs sham, and the bottom panels indicate the respective voxel‐wise correlations between functional activation and predicted electric field. All intensities show a positive correlation (i.e., higher electric field predicted greater CBF increase relative to sham). Note that higher intensities of 1.5 and 2.0 mA showed a stronger association. (c) Correlation between predicted electric field strength and tDCS‐induced CBF changes as a function of current intensity for cathodal‐M1 tDCS. All intensities show a negative correlation (i.e., higher electric field predicted greater CBF decrease relative to sham). Note that the 1.0 mA intensity showed the strongest association. CBF, cerebral blood flow; tDCS, transcranial direct current stimulation

We also performed exploratory analyses to cross‐validate the association between physiological effects of tDCS and the accuracy of realistic physical models of the predicted changes in the electric field. A map of the electrode montage and location of the target electrode is presented in Figure 6a, alongside the distribution of the electric field norm (|E|) projected on to a gray matter‐segmentation of the MNI brain. As can be seen, the electric field ranges diffusely from the left M1 electrode area toward the contralateral frontal‐orbit return electrode, with the maximum peak distributed along the posterior ridge of the left precentral gyrus (Figure 6a). Spearman rank correlations were then calculated at the voxel level between the electric field model and the statistical maps of the grand‐average difference in CBF between each active intensity and sham. For anodal‐M1 tDCS (Figure 6b), strongest correlations were observed with 1.5 and 2.0 mA (Spearman rho = 0.295, *p* < .001 for 1.5 mA and Spearman rho = 0.204, *p* < .001 for 2.0 mA). In other words, for these intensities, voxels which were predicted to have greater electric field relative to other voxels in the cortex correlated with relatively higher and positive changes in CBF. 0.5 and 1.0 mA intensities did not show strong correlations, although the directionality of the correlation was positive. For cathodal‐M1 tDCS, the strongest correlation was seen with 1.0 mA cathodal tDCS, whereby voxels with higher predicted electric fields were associated with more negative/decreased values of CBF (Figure 6c). For the remaining intensities, directions of all correlations were also negative, but relatively weaker.

## 4 DISCUSSION

The present investigation focused on clarifying the dose–response relationship of anodal and cathodal tDCS on cerebral blood flow, and the association of cerebral blood flow with cortical excitability alterations. Based on previous TMS‐MEP studies in the field, we hypothesized that there would be a partially nonlinear relationship between the delivered current intensity with physiological measures of cortical activity. In practice, we observed evidence pointing to linear polarity and intensity‐dependent dose effects for both anodal and cathodal tDCS, which could be predicted by a biophysical, finite‐element analysis of the induced electric field of the stimulation montage. In the following, we discuss the results in more detail, and propose possible mechanisms on the basis of current and previous findings.

### 4.1 Polarity and intensity‐dependent effects of tDCS on local CBF

For all active intensities of anodal‐M1 tDCS, cerebral blood flow in the area directly underneath the target electrode (i.e., the left M1 hand knob) increased significantly relative to sham tDCS, both during stimulation, as well as up to 90–105 min after the end of stimulation. The highest intensity of 2.0 mA resulted in the greatest increase in CBF, as compared with the other 0.5–1.5 mA intensities. For effects of cathodal tDCS on left M1 hand knob CBF, all intensities showed a tendency for an initial increase in CBF during the stimulation block, which then gradually declined to levels lower than baseline and sham tDCS for the remaining course of the scanning session, where the highest intensity of 2.0 mA resulted in the greatest decrease in CBF. For both anodal and cathodal M1 tDCS, we observed that the overall change across all timepoints was linearly intensity‐dependent (*r* = 0.28, *p* = .014 for anodal M1 tDCS, and *r* = −0.31, *p* = .009 for cathodal M1 tDCS). Moreover, the polarity‐dependent directionality of the perfusion changes was regionally specific to the target area of the M1 electrode, as no clear modulation of CBF was observed in the control ROI region. Collectively, these findings would be supportive of a monotonic input–output function between induced electric field strength and cerebral blood flow (Zheng et al., 2011). It should also be noted that with respect to the target M1 electrode, the anode electrode induced greater changes in perfusion against baseline, as compared to the cathode across all active intensities, suggesting a slightly superior effect of the anode electrode, as predicted by animal and computational models (Lafon, Rahman, Bikson, & Parra, 2017). Moreover, when the anodal electrode served as the return electrode over the right prefrontal region (i.e., cathodal M1 tDCS), intensities of 0.5 and 1.0 mA did not effectively alter perfusion, whereas higher intensities of 1.5 and 2.0 mA induced early increases in CBF. Besides supporting the evidence for a CBF‐enhancing effect of the anode electrode over the prefrontal cortex, this finding in particular demonstrates that at high current intensities, the return electrode should not necessarily be considered as physiologically inert, given the immediate increase in perfusion. These changes at the prefrontal site could potentially even result in behavioral effects (Nitsche et al., 2007). Lower intensities up to 1.0 mA, however, do not appear to induce any effective alterations in perfusion, which supports the conclusion of a physiologically inert electrode in the previous study (Nitsche et al., 2007). Nevertheless, future studies should take this finding into consideration when designing electrode montages, since the role of the frontal‐orbit/prefrontal return electrode(s) is often assumed to be negligible, but in actuality may factor into a physiological and/or functional role depending on current intensity.

Our observations of polarity‐dependent blood flow alterations as a result of low‐intensity DC stimulation are in accordance with previous animal and human studies. In a tDCS rat study using fMRI, Takano et al. (2011) observed an increase in the fMRI signal across the frontal areas and nucleus accumbens when an anodal current of 400 μA was applied over the frontal cortex with the return electrode positioned over the neck. In a tDCS study on rats which measured CBF using laser Doppler flowmetry, Wachter et al. (2011) reported a polarity‐specific and intensity‐dependent effect, where applying anodal current through an epicranial electrode in the range of 25–100 μA, with the return electrode placed over the ventral thorax led to an increase in local perfusion at the site of the cephalic electrode, which remained stable for at least 30 min. On the other hand, cathodal tDCS led to antagonistic effects on perfusion. In a subsequent study, Mielke et al. (2013) applied higher intensities of cathodal tDCS (200–700 μA) and found regional and long‐lasting decrease in CBF for up to 90 min after stimulation. In human studies, converging evidence across several studies using different imaging techniques also lend to a consistent finding of increased CBF during and post anodal stimulation. A few studies have reported cerebrovascular effects using noninvasive transcranial Doppler (TCD) recordings, which measures changes in CBF velocity and vasomotor reactivity. Giorli et al. (2014) reported a 21% increase in CBF velocity after 1 mA anodal tDCS over the right M1, and a 9% decrease after cathodal tDCS. A few studies have also investigated oxyhemoglobin concentrations using functional near infrared spectroscopy (fNIRS), which can be considered mechanistically similar to fMRI, in that it relies on neurovascular coupling to infer changes in neural activity, although at a reduced spatial resolution (Hoshi, 2016). Merzagora et al. (2010) reported a significant increase in the concentration of oxyhemoglobin following 10 min of anodal tDCS, which also led to after‐effects with a delayed peak. A more recent fNIRS study in healthy humans by Muthalib, Besson, Rothwell, and Perrey (2018) also found elevated oxyhemoglobin levels in the ROI underneath the anodal M1 stimulation electrode, compared to an ROI that was outside the spatial extent of the target electrode. A similar finding was observed when Zheng et al. (2011) investigated CBF using ASL‐fMRI during and immediately following short durations of M1 tDCS at intensities ranging from 0.8 to 2.0 mA. Here a generally linear intensity dependent relationship of increased CBF around the target electrode ROI during and shortly after anodal tDCS did take place, while cathodal tDCS initially enhanced CBF during stimulation, but then significantly decreased it below baseline during recording of after‐effects. No effects were observed in a control ROI located over the right temporo‐occipital region. Although our present findings are in general agreement with the work of Zheng and colleagues, we note that their study may not be completely comparable with ours, because in the latter study, an alternating on–off–on paradigm was used to probe the effect of different intensities, and previous work has shown that homeostatic and interference mechanisms may affect synaptic plasticity when stimulation is intermittently repeated (Fricke et al., 2011; Monte‐Silva et al., 2013). Our results are also in general accordance to a previous ASL study by Stagg et al., who observed an increase of 2–3% in perfusion during and immediately after 20 min of 1.0 mA anodal tDCS over the left DLPFC (Stagg et al., 2013). The stronger effects observed here in the M1 region might be due to differences in receptor and neurotransmitter characteristics between these regions (Del Arco & Mora, 2009), the structural and functional architecture of the cortex (Miller, Freedman, & Wallis, 2002) and/or the different path of DC current flow into this region.

### 4.2 Comparison between CBF‐ and motor‐cortex excitability changes following tDCS

In order to determine the relationship between after‐effects in perfusion and current intensity‐ and polarity‐dependent neuroplastic changes in cortical excitability following anodal and cathodal‐M1 tDCS, we compared our findings with a TMS‐MEP study (Jamil et al., 2017), which included the subset of participants in the present study, as well as the same tDCS parameters (i.e., current intensity, polarity, and stimulation duration, as well as the same individualized positioning of the M1 electrode over the TMS hand motor‐hotspot area). Importantly, including subjects who took part in the previous study allowed us to calculate within‐subject correlation coefficients between respective changes in CBF and cortical excitability.

At the overall group level, we found that tDCS‐induced changes in motor cortical excitability were not completely polarity or intensity‐dependent. Although all intensities of anodal M1 tDCS resulted in an enhancement of cortical excitability, only 0.5 and 1.0 mA of cathodal tDCS resulted in a significant excitability diminution, with higher intensities resulting in no clear effects. In a *post‐hoc* analysis, we computed subject‐level correlations between CBF and MEP alterations, separately over an “early” and “late” time bin in order to account for the observed dynamics in cortical excitability changes. For anodal M1 tDCS, we found significant positive correlations with both the left sensorimotor network (SMN) and left M1 electrode region for 1 mA anodal M1 tDCS, and a trend for a correlation with the left SMN for 0.5 and 1 mA cathodal M1 tDCS intensities, although these did not survive multiple comparison correction. These results denote that effects of tDCS on cortical excitability were of a similar magnitude as the effects of tDCS on CBF per individual. For anodal‐M1 tDCS, the correlation for 1.0 mA was significant in the second 60 min of the scanning period, for both the SMN ROI and the hand knob ROI. Although we observed less stronger correlations with other intensities, we found that excitability after‐effects of all anodal and cathodal tDCS intensities were in the same direction as CBF changes in the left SMN ROI (Figure 5c). This finding suggests that alterations of tDCS on cortical excitability as measured using TMS may be more comparable with hemodynamic changes when the functionally and anatomically connected network is considered as a whole, as opposed to the specific hand knob region by itself. In previous studies which have probed the cortical and subcortical circuits underlying the TMS‐induced motor‐evoked potential, premotor and parietal regions have been conclusively shown to influence motor‐cortico‐spinal excitability (Bestmann & Krakauer, 2015; Reis et al., 2008). Moreover, tDCS using smaller target electrodes than ours and applied to the premotor or parietal cortex was shown to significantly affect TMS‐induced motor cortex excitability measures (Boros, Poreisz, Münchau, Paulus, & Nitsche, 2008; Rivera‐Urbina et al., 2015). In a recent study, when the target electrode of the same size as ours was rotated to include areas of the premotor cortex, MEP alterations were superior (Foerster et al., 2018). Thus, the larger area covered by the rotated target electrode, which included not only the primary motor cortex, but also the premotor and somatosensory cortex, may have affected both excitability and perfusion activity of the inter‐connected sensorimotor network. This finding would support the rationale for performing network stimulation in order to more effectively enhance neuroplastic effects, as recently demonstrated by Fischer et al. (2017).

### 4.3 Comparison of fMRI‐CBF with TMS‐MEP as a physiological biomarker of tDCS after‐effects

Given that both the TMS‐MEP and ASL‐CBF findings demonstrate long‐lasting polarity‐ and intensity‐dependent physiological alterations, an intriguing question is which mechanistic process(es) constitute the physiological origin of the lasting perfusion effects, and whether these effects are directly linked to excitability changes. According to neurovascular coupling, the metabolic demand in response to changes in neuronal activity will affect local cerebral blood flow, which could be one basis for the effects observed here. As previously mentioned, one of the primary mechanisms underlying neuroplastic after‐effects of tDCS is the activity of NMDA receptors and calcium channels (Liebetanz et al., 2002; Nitsche, Fricke, et al., 2003). Through neurovascular coupling, postsynaptic glutamate acting on neuronal NMDA receptors has been shown to modulate cerebral blood flow, primarily through the Ca^2+^ dependent release of nitric oxide (NO), which subsequently acts on cyclic guanosine monophosphate (cGMP), resulting in arterial vasodilation, or secondarily through conversion of arachidonic acid to prostaglandins, which also dilate vessels (Attwell et al., 2010). Thus, membrane depolarization combined with an increase in spontaneous activity could lead to increased probability of NMDA receptor opening, which would lead to increased calcium influx resulting in LTP on the one hand, and an increased probability of calcium dependent release of NO resulting in vasodilation on the other. However, neurovascular coupling cannot account for the complete results, as the disparity in the reverse/null pattern in excitability changes following high‐intensities of cathodal M1 tDCS would need to be explained. One candidate explanation here could be the bi‐directional effects of calcium flux, as observed in previous animal studies, where low postsynaptic calcium induced long‐term depression, while larger calcium concentrations induced long‐term potentiation (Cho, Aggleton, Brown, & Bashir, 2001; Lisman, 2001). In contrast, no direct effect of neuronal calcium ion flux on regulation of cerebral blood flow has been so far made clear (Edvinsson et al., 1983). Alternatively, the observed nonlinear effects of cathodal tDCS on excitability could be due to activation of neuronal populations in deeper layers of the cortex, as a result of the increased electric field strength. This could potentially include activation of inter‐neuronal circuits, which play an important role in the homeostatic regulation of synaptic plasticity (Calcagnotto, 2016). Notwithstanding, further studies would be needed to understand the extent to which neurovascular (un‐)coupling may depend on increasing current intensities.

If the observed effects are not completely accounted for by neurovascular coupling, potentially these effects could also be partially driven by direct effects of stimulation on blood vessels. Direct vessel dilation has been proposed as potential mechanism in previous studies given the possibility that low intensities of DC stimulation may exert direct effects on endothelial cells which can increase nitric oxide production (Trivedi, Hallock, & Bergethon, 2013). Both anodal and cathodal tDCS induced a relatively small increase in CBF during the online/stimulation block of the scan. Thus, we cannot completely rule out the possibility of vessel dilation effects during stimulation, however these would not be expected to account for the polarity‐dependent after‐effects observed here, considering that cathodal tDCS ultimately resulted in a CBF decrease persisting for up to the end of the 2 hr session. In accordance, in the previously mentioned intact animal study of Wachter et al. (2011), the authors hypothesized that given the symmetric nature of the polarity‐dependent effects, lasting after‐effects in cerebral perfusion were more likely to be due to neurovascular coupling in contrast to direct vessel effects (Wachter et al., 2011). Further evidence for a neuronal origin of tDCS effects can be ascertained from a study on animal slice preparations in which circulation was absent, and synaptic plasticity was still observed (Fritsch et al., 2010; Pulgar, 2015).

A second explanation for the observed association between cortical excitability and vascular effects of tDCS besides neurovascular coupling could be astrocytic activity, which is also affected by tDCS (Ruohonen & Karhu, 2012), and has been shown to play a role in synaptic plasticity (Takata et al., 2011) as well as in the regulation of blood flow (MacVicar & Newman, 2015). In accordance, an animal study using calcium imaging demonstrated changes in astrocytic activity after tDCS (Monai et al., 2016). The interacting role of glial, as well as pericytic cells on tDCS‐induced neuroplastic changes is an unexplored topic and would benefit from further investigation.

Alternative explanations that changes in M1 CBF were not due to the specific electrode montage, but generated by skin sensations, or nonspecific effects such as changes of the autonomous nervous system, heating of the electrodes or changes in arousal state are unlikely. For anodal‐M1 tDCS across all intensities, no immediate and marked effects in perfusion changes were observed in the contralateral return electrode region, which would be expected in case of polarity‐independent localized temperature changes through the skin (Khadka et al., 2018; Nitsche & Paulus, 2000). Likewise, no polarity‐ or intensity‐dependent changes were observed in the control region, which would be expected in case of a nonspecific increase in perfusion based on an autonomous response. Moreover, any potential changes in arousal state or other nonspecific effects would also be reflected in the sham condition, which, for both anodal and cathodal tDCS, remained at levels close to baseline for the major portion of the scanning. A drift in CBF toward the end of the scanning was however observed across all conditions independent of tDCS intensity or polarity, which we suspect to be due to a normal decline in attention (Oken, Salinsky, & Elsas, 2006).

### 4.4 Predictive value of biophysical electric field model and anatomical covariates

As a *post‐hoc* analysis, we assessed whether the physiological effects of tDCS across the whole brain correlate with the predicted electric field. By comparing the group‐level statistical changes in CBF at the voxel level with the modeled results of our electrode montage, we could derive a coarse estimate of how well the model predicted the physiological results at a high spatial resolution. For anodal‐M1 tDCS, a high positive correlation between predicted electric field and change in CBF against sham tDCS was observed at the higher intensities of 1.5 and 2.0 mA (in conventional estimates, corresponding to 8.70 and 4.16% of the explained variance, respectively), while for cathodal‐M1 tDCS, a significant negative correlation was only observed for 1.0 mA (4.84% of explained variance). We note that the directionality of the respective correlations of the remaining intensities conformed to a negative direction for cathodal‐M1 tDCS and positive for anodal‐M1 tDCS. Although these correlations may appear weak, it is important to note that more accurate predictions could principally be achieved if models were individualized to each subject's head, which was not within the scope of the current study. Nevertheless, our findings are compatible with those of a previous study which employed individualized modeling to validate fMRI effects in a single patient (Halko et al., 2011). Previous studies attempting to validate physiological effects with predicted electric fields have found better accuracy with intra‐cranial measurements (Huang, Liu, et al., 2017; Opitz, Falchier, Linn, Milham, & Schroeder, 2017), where current flow changes can be read out directly with implanted electrodes. In this way, invasive EEG approaches may yield better accuracy of the electric field model, in addition to better temporal resolution, but weaker spatial resolution as compared with fMRI, due to inherently limited electrode coverage. Thus, resolving the optimal parameters for assessing physiological changes correlated with the induced electric field in the brain remains an open challenge.

In another *post‐hoc* analysis, we investigated sources of inter‐individual variability in our dataset (Supporting Information 3). No relationship between after‐effects and TMS sensitivity was found, as reported in previous cortical excitability studies (Jamil et al., 2017; Labruna et al., 2016). However, there was a small trend whereby an individual's M1 gray matter volume tended to correlate with efficacy of 2.0 mA anodal‐M1 tDCS. The explanation for this finding is unclear. It could be possible that greater volume of gray matter may impact the electric field conductivity causing greater effects (Miranda, Mekonnen, Salvador, & Ruffini, 2013). This finding may hold implications for gray‐matter related variances in clinical subgroups, where brain atrophy may affect current distribution (Mahdavi & Towhidkhah, 2018). Further studies to incorporate these metrics in individualized computational models to determine their physiological relevance is a desirable goal.

### 4.5 Limitations and future directions

In light of the exploratory nature of our study, it is important to mention some limitations. First, regarding methodology, we included only young healthy adults between ages of 18–45, who were right‐handed and nonsmokers. These subjects were re‐enrolled after already participating in five sessions of TMS‐MEP experiments with the same tDCS parameters, and thus, they were no longer naïve to stimulation. However, any discussion or disclosure of stimulation parameters was avoided by the experimenters until the end of the entire study. In addition, although subjects were blinded to the stimulation to reduce bias, the experimenters delivering the tDCS were not able to be blinded due to practical limitations. Moreover, the sample size of 29 subjects was also a practical limitation, given the long (~3 hr) duration of a single scanning period, multiple scanning sessions, and exploratory nature of the study design. To increase statistical power, we used a repeated‐measures design, whereby each participant also took part in a control condition, which reduces the effect of inter‐subject variability in the findings. Thus, we believe our findings should provide adequate implications, irrespective of the moderate sample size, but further studies with larger samples would nevertheless be ideal. Another limitation to be noted is that the long duration of scanning may have led to drifts in the participant's arousal and attention levels, which is commonly observed in long‐duration EEG recordings (Oken et al., 2006). This can be seen also by a drift in CBF in the sham tDCS condition. To mitigate the effect of arousal flux, we engaged with participants in between blocks through a bidirectional intercom system and no indications of sleep, or transitions to sleep, were noticed through the course of the scanning. Participants also used a response pad to self‐report their level of tiredness on a Likert scale, which did not differ by stimulation condition or time (all values of *p* > .05). Nevertheless, future studies employing longer duration scanning protocols may consider simultaneously recording EEG in order to monitor state changes. Regarding interpretation of the findings, we cannot conclude whether the effects reported will hold during nonresting state activities, such as during learning or performance of a motor activity, given that stimulation‐induced plasticity may be state‐dependent (Antal, Terney, Poreisz, & Paulus, 2007). Moreover, whether longer stimulation or higher current intensities would result in greater effects is an intriguing question which cannot be directly concluded or extrapolated from this study, considering the nonlinearity of the stimulation duration, and intensity parameters (Batsikadze et al., 2013; Monte‐Silva et al., 2010; Monte‐Silva et al., 2013), and the precaution that subject blinding becomes more difficult with higher stimulation intensities (Woods et al., 2016). Finally, regarding transferal of these findings, it is important to note that when considering the heterogenous spatial distribution of the arterial supply and vascular tone across the cortex (Fan, Jahanian, Holdsworth, & Zaharchuk, 2015), as well as spatial differences in neurotransmitter receptor densities (Zilles, Palomero‐Gallagher, & Schleicher, 2004), it is not self‐evident that the observed characteristics of the physiological effects observed by tDCS on the M1 will transfer one‐to‐one to other cortical regions, and further studies would be needed to test this assumption. Likewise, given the potential impact of the current results to clinical populations, the findings presented here in young healthy adults cannot be assumed to transfer one‐to‐one with patient brains, or older adults, where an age‐related reduction in steady‐state CBF and cerebral metabolic rate is well known (Tarumi & Zhang, 2018). Parallel investigations across these additional populations will thus be informative for determining the therapeutic efficacy of tDCS. Besides exploring the impact of tDCS on other populations, more focus is also needed to determine how inter‐individual differences in anatomy might impact the distribution of the electric field, and thereby also influence the physiological response. In this view, we were limited to modeling only the response of tDCS at the group level, but a more nuanced approach which considers the individual anatomical differences is a topic of ongoing work.

## 5 CONCLUSION

In the present study, we investigated the effects of anodal and cathodal tDCS on regional cerebral blood flow, both during and up to 120 min after stimulation. For anodal‐M1 tDCS, we observed an intensity‐dependent linear increase in CBF, such that 0.5–1.5 mA intensities of anodal‐M1 tDCS resulted in relatively modest CBF increases (3–5%) in the targeted the left primary motor cortex, which returned to baseline after 60–75 min while 2.0 mA anodal‐M1 tDCS resulted in the greatest CBF increase, which lasted the entire 2 hr scanning duration. Cathodal‐M1 tDCS intensities of 1.0 and 2.0 mA resulted in decreased perfusion compared to sham tDCS, which was also present up to the end of the 2 hr monitoring. Moreover, we observed correlations between changes in CBF in the sensorimotor region and motor‐cortical excitability measured using TMS‐MEP at the individual level. These findings indicate that application of weak currents to the resting state cortex not only alters cortical excitability, but also leads to prolonged changes in cortical perfusion, which appear to span for at least 2 hr.

## CONFLICT OF INTEREST

M.A.N. is a member of the advisory board of Neuroelectrics.

## Supporting information


**Appendix S1**: Supporting Information

## Data Availability

The data that support the findings of this study are available on request from the corresponding author. The data are not publicly available due to privacy or ethical restrictions.
