# Supplementary material for: Current intensity‐ and polarity‐specific online and aftereffects of transcranial direct current stimulation: An fMRI study
Source: Hum Brain Mapp. 2019 Dec 20;41(6):1644–66. doi: 10.1002/hbm.24901 (PMC7267945; doi:10.1002/hbm.24901)
Supplement: Supplementary file 1 — Appendix S1: Supporting Information [file HBM-41-1644-s001.docx]

Supplementary Information/Figures

# Whole-brain analysis of CBF modulation

We performed a whole-brain analysis to investigate the influence of stimulation polarity and current intensity on perfusion changes across the entire brain. First, for each session, the subject’s 4-dimensional perfusion volume for each scanning block was entered into a first-level fixed effects model to obtain nine *T*-contrasts representing the within-session difference between the baseline (pre-tDCS) and post-stimulation perfusion (i.e., each map representing the stimulation-induced after-effects of the individual at the particular timed measurement point). These nine contrasts for each of the five sessions (a total of 45 contrasts per subject) were entered into a second-level analysis, using SPM12's flexible factorial model to contrast main effects of Intensity, Time, and respective interaction effects. We modeled *F*-contrasts for the two main effects and the interaction, as well as *T*-contrasts (two-tailed) to contrast the overall effect of an active-tDCS intensity (0.5 – 2.0 mA) against sham tDCS. Follow-up tests for the Time factor were again assessed by pooling the time course into two time-bins, 0-60 min and 60-120 min. For all comparisons, inference testing proceeded after contrast images were corrected for multiple comparisons by means of SPM12’s FWE correction.

**Anodal-M1 tDCS:** A whole-brain analysis revealed widespread, spatially distributed effects from all active intensities of anodal tDCS. Using *F-*contrasts, we first evaluated the effects of the experimental factors of Intensity, Time, and Time x Intensity interaction. For the factor Intensity, several significant activation clusters were identified (Figure 1A), the strongest being the left anterior cingulate (peak: F(4,616)=26.068, cluster p=0.001, FWE corrected; Figure 1B). For the factor Time, the *F*-contrast identified a very large number of significant clusters; the peak of the major activations appeared to be located bilaterally in prefrontal and frontal areas, including the frontal poles, as well as dorsal parts of the precuneus and cingulate cortices (Figure 1C). An Intensity x Time interaction contrast did not reveal any significant clusters. To assess intensity-wise effects, we conducted two-tailed paired *t*-tests to contrast the overall main effects of the active intensity against the overall main effect of the sham condition. The main differences are reported in Figure 1D and can be summarized as follows: after FWE correction for multiple comparisons, a cluster-wise analysis for 0.5 mA anodal tDCS yielded significant activations in the right superior frontal gyrus (peak: T(616)=7.534, p=<0.001) and the left precentral gyrus (peak: T(616)=5.648, p=<0.001)(Figure 1D.1). For 1.0 mA, we observed two clusters of increased activation (Figure 1D.2): the first located in the left anterior cingulate gyrus (peak: T(616)=9.173, p=<0.001 FWE corrected) and a second located in the left Heschl’s gyrus (peak: T(616)=8.253, p=<0.001 FWE corrected). For 1.5 mA, a significant cluster of *decreased* CBF relative to sham was observed in the left frontal pole (peak: T(616)=5.799, p=<0.001), while increased CBF was observed in the left midcingulate (peak: T(616)=7.293, p=0.001) and left Rolandic Operculum (peak: T(616)=6.933, p=0.001 FWE corrected; Figure 1D.3). For 2.0 mA, activation was observed across a large portion of the frontal cortices (Figure 1D.4). The center peak of these clusters were identified around the left middle frontal gyrus (peak: T(616)=8.588, p=0.001 FWE corrected), left superior frontal gyrus (peak: T(616)=8.276, p=0.001 FWE corrected) and the right middle frontal gyrus (peak: T(616)=4.259, p=0.001).


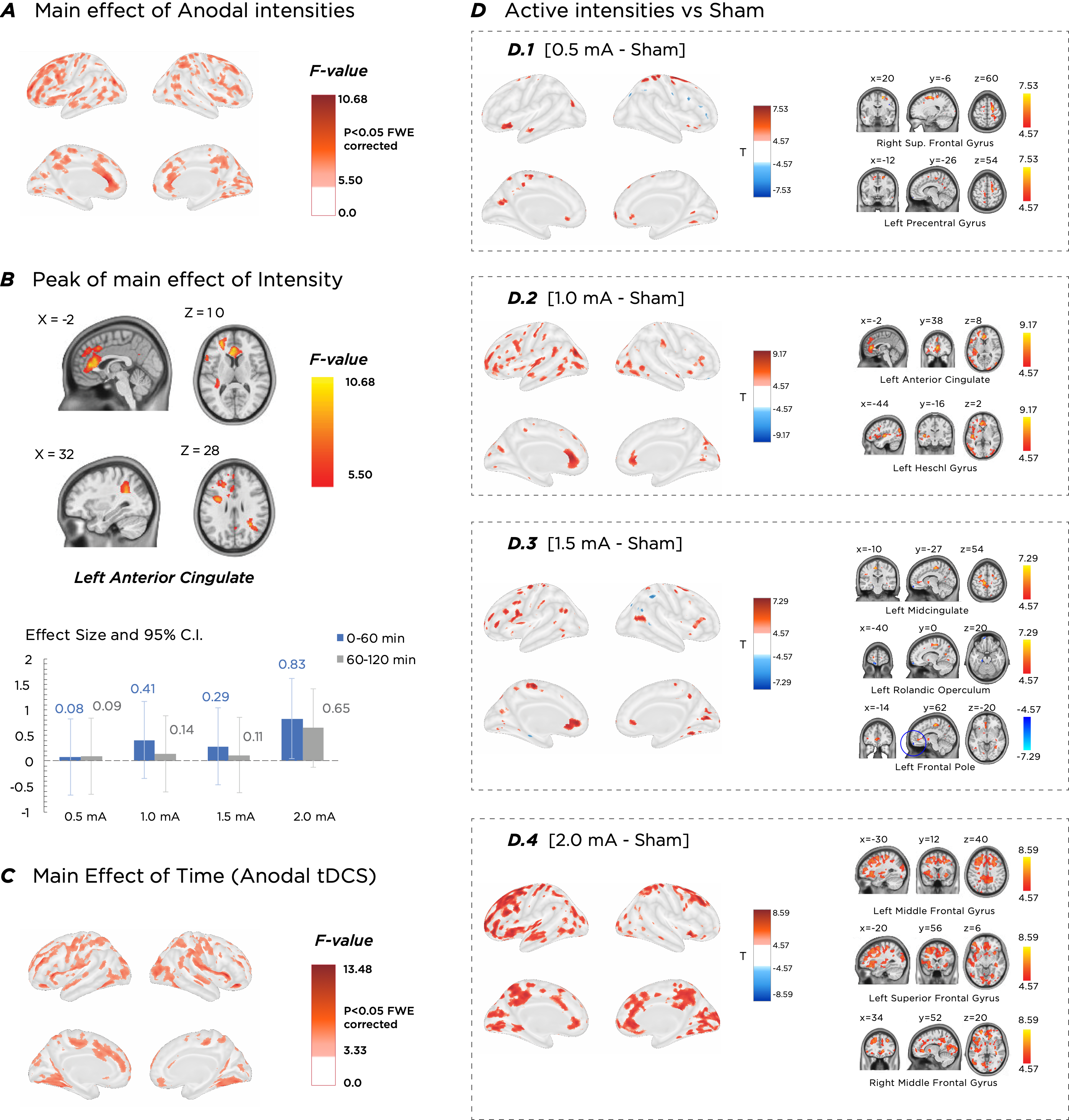


**Figure 1: Anodal tDCS-induced modulation of perfusion activity**. For contrasts of main effects (panels A and C), darker pink to red coloring indicates higher critical values (p<0.05 FWE corrected). A) A main effect of anodal tDCS intensities revealed widespread activation predominantly over frontal areas, but also including posterior areas. B) Peak activations were identified in the left anterior cingulate gyrus. Follow-up calculations of parameter effect size estimates on this cluster revealed the greatest effect relative to sham with 2.0 mA, followed by 1.0 and 1.5 mA, and then 0.5 mA. This pattern held for both time-bins. C) Effect of Time on CBF, averaged over all anodal stimulation intensities. Activations generally appeared widespread, spanning across prefrontal, temporal and parietal areas. D) Paired contrasts between active anodal tDCS intensities and sham tDCS, averaged over time. Blue colors indicate a negative difference and red colors indicate a positive difference. Relative to other intensities, 0.5 mA showed smaller dispersed activations while 1.0, 1.5 and 2.0 mA resulted in widespread activations, mainly in the left hemisphere. Decreased CBF relative to sham was observed in the left fronto-polar region for 1.5 mA anodal-M1 tDCS, while 2.0 mA induced the widest (spatial) extent of CBF increase, with the center peaks spanning across the left frontal regions.

**Cathodal-M1 tDCS:** *F­*-contrasts for main effect of tDCS Intensity revealed clusters of activation over frontal, temporal and parietal areas (Figure 2A), with the maximum peak located frontally in the left superior temporal gyrus (peak: F(4,572)=17.751, p=<0.001 FWE corrected). Similar to anodal tDCS, the main effect analysis for the factor Time showed significant modulations throughout frontal and parietal areas (Figure 2B). An Intensity x Time interaction contrast (Figure 2C) also revealed a significant cluster located in the right frontal pole (peak: F(32,572)=2.98, p=0.009 FWE corrected; Figure 2D). Parameter estimates of this cluster were contrasted between each active intensity and sham across 60-min time-courses. Here, higher intensities of 1.5 and 2.0 mA only during the first 60 min period resulted in greater effects compared with lower intensities as well as with the final 60-120 min period. In subsequent assessments for the main intensity-wise differences to sham (Figure 5E), paired *t*-contrasts between 1.0 mA cathodal tDCS and sham revealed a cluster of decreased CBF relative to sham in the left Insula Lobe (peak: T(572)=6.446, p=<0.001 FWE corrected), left middle temporal gyrus (peak: T(572)=5.856, p=<0.001 FWE corrected), as well as a decreased CBF cluster near the left frontal pole (peak: T(572)=5.383, p=<0.001, FWE corrected). Cathodal tDCS at 1.5 mA resulted in an increased CBF cluster in the right frontal pole (peak: T(572)=4.93, p=0.034 FWE corrected). For 2.0 mA, an increased CBF cluster was observed in the left frontal pole region (peak: T(572)=5.320, p=0.027 FWE corrected) and a decreased CBF cluster in the left Insular Lobe (peak: T(572)=6.709, p=<0.001 FWE corrected) and right midcingulate (peak: T(572)=6.075, p<0.001 FWE corrected).


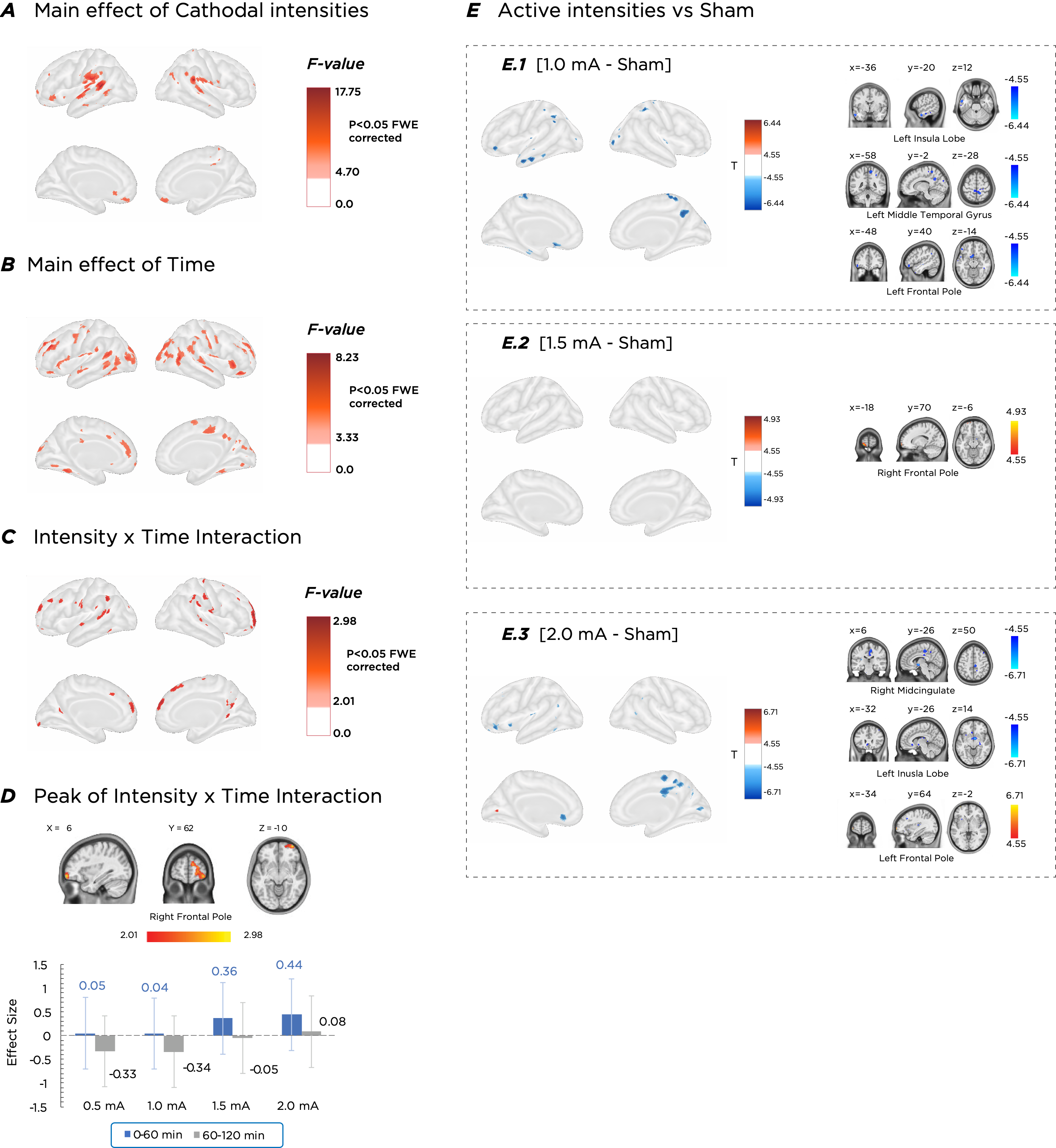


**Figure 2: Cathodal tDCS-induced modulation of perfusion activity**. For contrasts of main effects (panels A, B and C), darker pink to red coloring indicates higher critical values (p<0.05 FWE corrected). **A)** A main effect of cathodal tDCS intensities revealed a maximum peak in the right paracingulate cortex, with further activation proximal to bilateral temporo-parietal areas, as well as in frontal regions. **B)** Effect of time on CBF, averaged over all cathodal stimulation intensities. Activations resembled the counter-part pattern with anodal tDCS (Figure **4C**), which appeared widespread, spanning across prefrontal, temporal and parietal areas. **C)** An Intensity x Time interaction contrast revealed a cluster of activation between the right frontal pole region and the left and right frontal and parietal areas. **D)** Peak activation of this interaction was detected in a cluster spanning the right frontal pole, with effect size estimates higher for the 1.5 mA and 2.0 intensities vs sham. **E)** Paired contrasts between active cathodal tDCS intensities and sham tDCS, averaged over time. Blue colors indicate a negative difference and red colors indicate a positive difference. While no effects were observed for 0.5 mA, both 1.0 and 2.0 mA intensities resulted in decreased CBF relative to sham in left and right posterior and frontal regions. Moreover, both 1.5 mA and 2.0 mA resulted in increased CBF in the fronto-polar areas**.**

# Within-subject correlations between TMS-MEP and fMRI-CBF changes


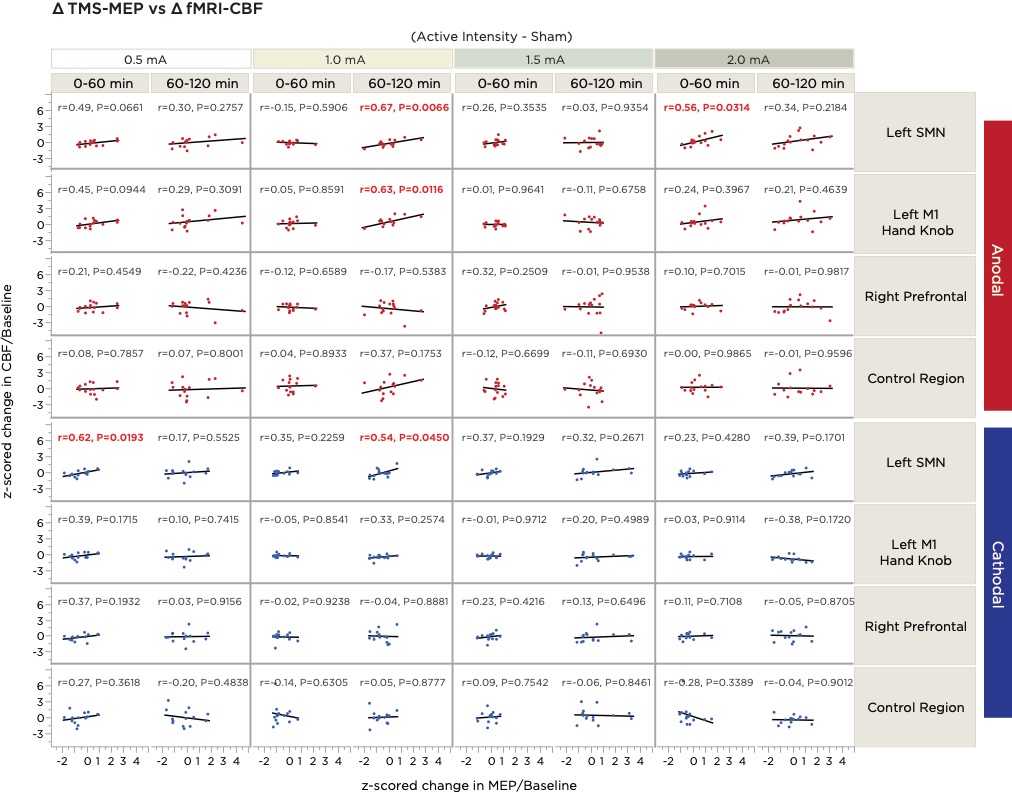


Figure 3 **Correlation analyses between time-binned averages in motor cortical excitability and cerebral blood flow**. MEPs were compared to CBF values from four regions of interest: the left sensorimotor network consisting of the primary and premotor cortices, the anatomical region underneath the target electrode, corresponding to the hand knob area; the anatomical region underneath the return electrode, corresponding to the right prefrontal region, and a control region away from both electrode sites, corresponding to the right temporo-occipital region. Correlations were calculated using Pearson correlation and coefficient values are presented above each comparison. Bolded red text indicates a significant correlation (p<0.05).

# Correlation between inter-individual anatomical covariates and tDCS effects

As an exploratory analysis, we assessed factors that could affect the inter-subject variability of the tDCS after-effects, which remains an important issue. We identified five relevant subject-specific markers for a covariate analysis. We first assessed whether an individual’s average TMS stimulus intensity required to generate a 1 mV MEP amplitude in the ADM muscle (SI_1mV_, approximately 130% of the resting motor threshold) might have been a factor that could determine tDCS efficacy, as it was recently identified in a multi-study analysis of cortical excitability effects [Labruna et al., 2016]. Additionally, we investigated whether the distance between the scalp area of the electrode to the center of the motor cortex may have been relevant in affecting the induced physiological changes of tDCS [Opitz et al., 2015]. Third, we investigated whether an individual’s total gray matter volume (GMV) in the left M1 may have led to variable effects in CBF, potentially due to variable structural integrity [Boes et al., 2017]. Fourth, we investigated whether the age of an individual may have been functionally relevant [Datta et al., 2012; Fujiyama et al., 2014], and lastly, we investigated whether a participant’s exact CBF at baseline may have influenced the extent of the intervention’s after-effect. The five covariates of interest were obtained as follows. SI_1mV_ was obtained as for each subject as the average intensity, measured as a percentage of the maximum stimulator output (% MSO), over the course of five sessions, and averaged together (note that this mean metric was considered reliable as the intensities did not significantly differ between sessions- SD=1.99, p>0.05). The electrode to cortex distance (ECD) was obtained individually per subject, based on anatomical segmentations. Briefly, the, high resolution T1 image from each subject was reconstructed as a 3D isosurface of the scalp, which allowed for the identification of the stereotaxic center of the target electrode on the scalp (see Figure 1C; reconstruction implemented in MRIcron - available open source at https://www.nitrc.org/projects/mricron). The respective T1 volume was then processed using Freesurfer (<http://surfer.nmr.mgh.harvard.edu/>) for segmentation as well as a high-resolution parcellation of prominent cortical areas, based on cytoarchitechtonic landmarks [Fischl et al., 2004]. The left motor cortical region, Brodmann area 4 (BA4) was parceled as an ROI, and a Matlab script was used to calculate the 3D Euclidean distance between the center point of the electrode and the center point of the M1 ROI. The third metric—gray matter volume of the left M1—was also obtained from the Freesurfer-generated segmentation, which we used to generate a volume and morphometry report. Importantly, this function in Freesurfer attains greater accuracy than standard segmentation techniques due to additional surface based calculations, tessellation of the gray matter/white matter boundary, and automated topology correction [Fischl et al., 2001; Segonne et al., 2007]. As we collected individual anatomical images five times for each subject, the ECD and GMV measurements were calculated separately with each session’s high resolution T1 image and then averaged together. Finally, at the group level, the subject-specific metrics were first inspected for application of standard parametric analyses by means of Kolmogorov-Smirnov goodness of fit tests, and then correlated against the individual’s averaged post-stimulation CBF response extent, calculated as the grand average difference from 0-120 min post-tDCS. Thus, we obtained a correlation coefficient for each tDCS intensity to the five factors of interest. As this analysis was of exploratory nature, significance was inferred from an uncorrected alpha of 0.05. In order to assess the effect size of these covariates within the sample at hand, the 95% confidence intervals of these correlation coefficients were calculated based on a sample-wise bootstrap resampling procedure (1,000 Monte-Carlo simulations). All statistical calculations were conducted using the Statistics Toolbox for MATLAB (version 2015b, MathWorks Inc.) and SPSS (v22.0, IBM Corp).

Intensity- and polarity-dependent relationships of the five inter-individual covariate factors to CBF after-effects induced as a result of anodal and cathodal tDCS were assessed by correlation calculations. Respective findings are reflected graphically by means of Manhattan plots of critical values and confidence interval estimates of correlation strength (Figure 4A-B). For anodal-M1 tDCS, individual M1 GMV was found to correlate with effects of 2.0 mA tDCS (r=0.583, p=0.023; Figure 4A). Interestingly, this factor also correlated *negatively* with effects of both anodal and cathodal sham tDCS (r=-0.562 and -0.585, p=0.029 and 0.028 respectively). For cathodal-M1 tDCS, a correlation was also found between ECD and effects of 1.5 mA (r=-0.552, p=0.041; Figure 4B). No correlations were observed with baseline CBF, SI_1mV_, and age for either anodal- or cathodal-M1 tDCS. In additional analyses, we assessed the correlation of the motor cortex parameters (SI_1mV_, GMV, and ECD) at the individual level, independent of tDCS effects. Here, we found a close coupling between a participant’s SI_1mV_ and the ECD (r=0.596, p<0.001; Figure 4D), which corroborates previous findings in TMS literature demonstrating a functional relationship between motor threshold and the distance of the coil to the cortex [Herbsman et al., 2009; Kozel et al., 2000; McConnell et al., 2001]. Moreover, SI_1mV_ also correlated positively with GMV (r=0.531, p<0.001; Figure 4C), but GMV did not correlate with ECD (r=0.288, p=0.13; Figure 4E).


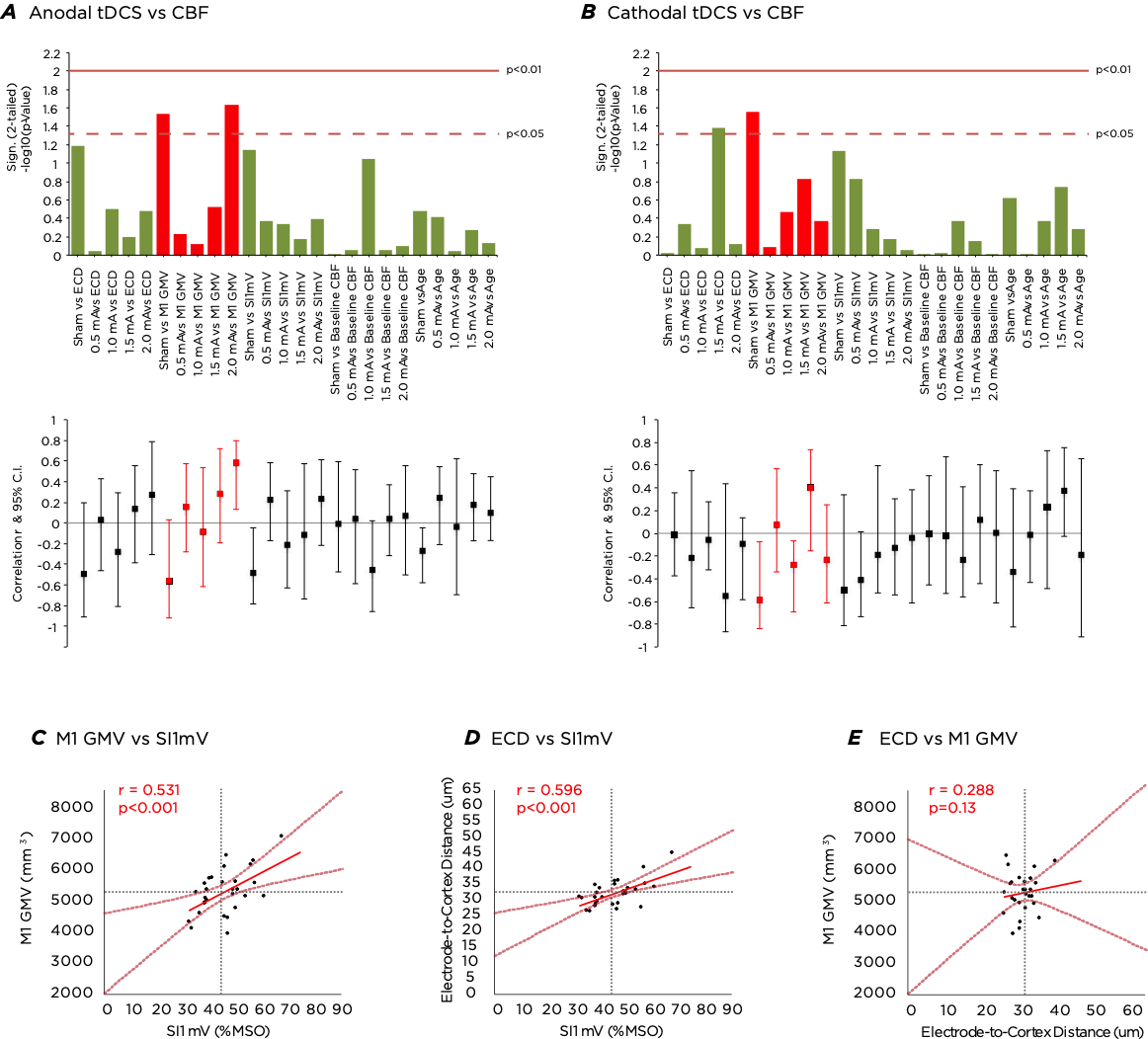


**Figure 4: Influence of individual covariates on tDCS after-effects.** In a series of regression analyses, after-effects of tDCS were compared to individual factors of age, baseline rCBF, gray matter M1 volume (GMB), electrode-to-cortex distance (ECD) and TMS sensitivity, measured as the stimulus intensity needed to elicit a 1 mV motor evoked potential of the right abductor digiti minimi muscle (SI1mV). The extent of the correlations is displayed as Manhattan plots and effect sizes. A) Anodal tDCS: gray matter volume was found to positively correlate with efficacy of 2.0 mA anodal tDCS (p<0.05, uncorrected). Moreover, there was a tendency of this factor to linearly depend on the intensity of tDCS (correlation points marked in red). Other factors had no relationship. B) Cathodal tDCS: There was no clear relationship between active tDCS intensities and individual covariates with after-effects in rCBF. In an overall analysis, gray matter volume and electrode-cortex distance was found to be closely correlated with SI1mV (C,D), while there was no relationship between electrode-cortex distance and gray matter volume (E).

# Threshold-based correlation analysis between predicted electric field and physiological effects of tDCS

As a confirmatory analysis to assess the strength of the correlation between the predicted electric field strength as modeled using FEM vs. the physiological effects of tDCS, we conducted a second comparison based on a subset of voxels which were most impacted by the tDCS electric field. In order to take into account the varying current intensities of the electric field model (which follows a linear pattern), we first determined a threshold of the electric field map based on the top 10% percentile of the |E| distribution for the 1.0 mA intensity, which resulted in a minimum electric field value of 0.25 V/m. We then applied this 0.25 V/m threshold for each of the four electric field models at the four intensities (e.g., 0.5, 1.0, 1.5, and 2.0 mA), which resulted in a gradient of increasing |E| from the lowest to the highest intensity, as shown in Figure 5A. We compared these thresholded |E| maps to the statistical fMRI maps of significant activation of each intensity relative to sham (for details, see section 1 above; significant clusters shown in panels B and C). The comparisons were quantified by calculating the Dice coefficient of similarity for both contrasts (i.e., increase and decreased activation relative to sham tDCS). As seen by the results of these comparisons (panels D and E), for anodal-M1 tDCS, a moderate degree of overlap was observed between the statistically determined clusters of CBF activation vs the predicted electric field (in the range of 10-25% agreement, similar to the whole brain correlation). For cathodal-M1 tDCS, the overlap was smaller and only for the 1.0 and 2.0 mA intensities, in the range of 2-5%.


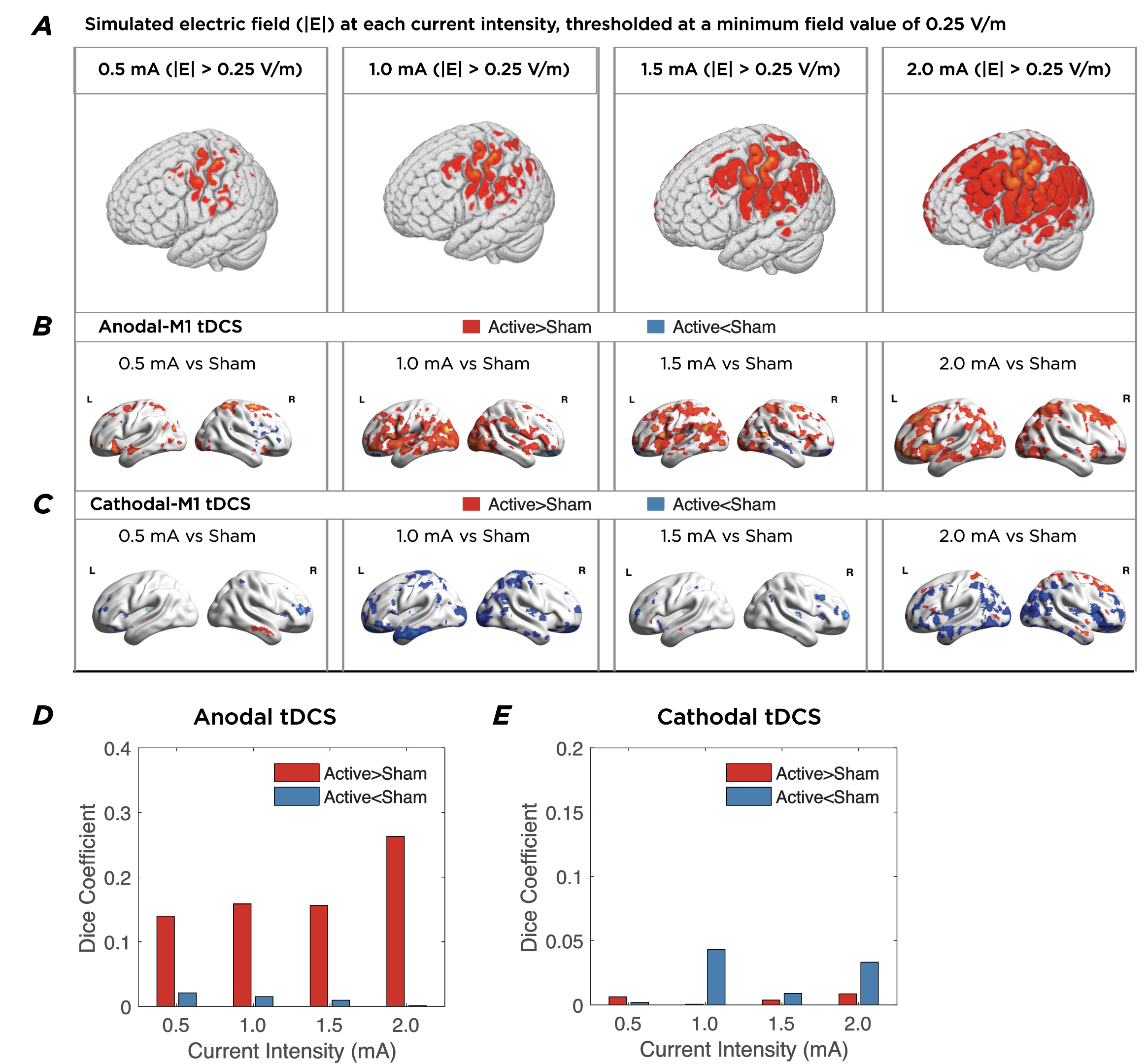


Figure 5

References

Boes A, Weigand A, Lan M, Liston C, Pascual-Leone A, Dubin M, Fox M (2017): 936. Cortical Thickness as a Biomarker of Repetitive TMS Treatment Response in Depression. Biol Psychiatry 81:S379.

Datta A, Truong D, Minhas P, Parra LC, Bikson M (2012): Inter-individual variation during transcranial direct current stimulation and normalization of dose using MRI-derived computational models. Front Psychiatry 3:1–8.

Fischl B, Liu A, Dale AM (2001): Automated manifold surgery: constructing geometrically accurate and topologically correct models of the human cerebral cortex. IEEE Trans Med Imaging 20:70–80.

Fischl B, Van Der Kouwe A, Destrieux C, Halgren E, Ségonne F, Salat DH, Busa E, Seidman LJ, Goldstein J, Kennedy D, Caviness V, Makris N, Rosen B, Dale AM (2004): Automatically Parcellating the Human Cerebral Cortex. Cereb Cortex 14:11–22.

Fujiyama H, Hyde J, Hinder MR, Kim S-JJ, McCormack GH, Vickers JC, Summers JJ (2014): Delayed plastic responses to anodal tDCS in older adults. Front Aging Neurosci 6:1–9.

Herbsman T, Forster L, Molnar C, Dougherty R, Christie D, Koola J, Ramsey D, Morgan PS, Bohning DE, George MS, Nahas Z (2009): Motor threshold in transcranial magnetic stimulation: The impact of white matter fiber orientation and skull-to-cortex distance. Hum Brain Mapp 30:2044–2055.

Kozel FA, Nahas ZH, DeBrux C, Molloy M, Lorberbaum JP, Bohning DE, Risch SC, George MS (2000): How coil-cortex distance relates to age, motor threshold, and antidepressant response to repetitive transcranial magnetic stimulation. J Neuropsychiatry Clin Neurosci 12:376–384.

Labruna L, Jamil A, Fresnoza S, Batsikadze G, Kuo M-FMF, Vanderschelden B, Ivry RBRB, Nitsche MAMA (2016): Efficacy of Anodal Transcranial Direct Current Stimulation is Related to Sensitivity to Transcranial Magnetic Stimulation. Brain Stimul 9:8–15.

McConnell KA, Nahas Z, Shastri A, Lorberbaum JP, Kozel FA, Bohning DE, George MS (2001): The transcranial magnetic stimulation motor threshold depends on the distance from coil to underlying cortex: A replication in healthy adults comparing two methods of assessing the distance to cortex. Biol Psychiatry 49:454–459.

Opitz A, Paulus W, Will A, Thielscher A (2015): Anatomical determinants of the electric field during transcranial direct current stimulation. Neuroimage 109:2.

Segonne F, Pacheco J, Fischl B (2007): Geometrically Accurate Topology-Correction of Cortical Surfaces Using Nonseparating Loops. IEEE Trans Med Imaging 26:518–529.
